# Transcriptomic profiling of the sex-linked biological pathways of severe pulmonary arterial hypertension associated with endothelial cell caveolin-1 depletion and chronic hypoxia

**DOI:** 10.3389/fphys.2026.1794886

**Published:** 2026-05-08

**Authors:** Joseph W. Leasure, Samuel M. Lee, Kayla L. Yerlioglu, Maricela Castellon, Haibin Li, Andrés Fantauzzi, Sunny Chen, Sami B. Muslmani, Arnav Sharma, Jiwang Chen, Richard D. Minshall

**Affiliations:** 1 Department of Anesthesiology, University of Illinois at Chicago, Chicago, IL, United States; 2 Department of Pharmacology and Regenerative Medicine, University of Illinois at Chicago, Chicago, IL, United States; 3 Department of Research Resources Center Cardiovascular Research Core, University of Illinois at Chicago, Chicago, IL, United States; 4 Department of Medicine University of Illinois at Chicago, Chicago, IL, United States

**Keywords:** bone morphogenetic protein (BMP), caveolin-1 (CAV1), endothelial cells (ECS), Pulmonary Arterial Hypertension (PAH), differentially expressed genes (DEG), gene ontology (GO)

## Abstract

**Introduction:**

Pulmonary arterial hypertension (PAH) is distinguished by elevated blood pressure and vascular resistance in the arteries of the lungs. Patients with PAH demonstrate pulmonary vascular remodeling, wall thickening, and a high rate of morbidity due to right heart failure. Notably, while female patients are more likely to develop PAH, male patients suffer from higher morbidity rates after diagnosis. The molecular mechanism(s) underlying PAH development is poorly understood, though heritable PAH linked to mutations in bone morphogenic protein receptor 2 (*Bmpr2*) and caveolin-1 (*Cav1*) may provide novel insights into the disease’s pathophysiology.

**Methods:**

To interrogate this dynamic, we utilized a global *Cav1* knockout (Cav1-KO) mouse model (Cav1*^-/-^*) in conjunction with chronic hypoxia to induce symptoms of PAH as demonstrated by hemodynamic and ECHO cardiography recordings.

**Results:**

Both female and male Cav1^-/-^ mice in chronic hypoxia demonstrated elevated right ventricular systolic pressure (RVSP) of 48.49 mmHg and 47.78 mmHg respectively. Female knockout mice began dying earlier in hypoxic conditions (4 wks), though male mice showed greater total mortality by the end of the 8 wks of hypoxia. In addition to wildtype controls, we compared this knockout mouse to endothelial-specific *Cav1* reconstituted (Cav1-RC) knockouts and found that restoration of *Cav1* expression only in endothelial cells (ECs) is sufficient to ameliorate PAH symptoms, highlighting the importance of vascular Cav1 in maintaining pulmonary artery function. RNA-sequencing of the lungs revealed that *Cav1^-/-^* is associated with downregulation of biological process gene pathways involved in cilium assembly in normoxic conditions for both sexes. In hypoxic conditions, *Cav1* knockout in females leads to downregulation of bone morphogenetic protein (BMP) signaling, while male hypoxic *Cav1^-/-^* led to a significant increase in muscle cell development genes. Reconstitution of Cav1 in ECs leads to upregulation of immune signaling pathways, muscle cell development, and various cell differentiation pathways in both sexes; females showed a unique upregulation of cilia-related pathways, while males demonstrated increased BMP signaling.

**Discussion:**

These data indicate that muscle cell development, angiogenesis, cilia assembly, immune response, and BMP signaling pathways undergo sex-specific transcriptional regulation during PAH development that may underlie sex differences in PAH patient outcome.

## Introduction

Pulmonary hypertension (PH) is a chronic lung disease characterized by mean pulmonary arterial pressure (mPAP) above 20mm Hg (Ruopp and Cockrill, 2022; Maron, 2023). Pulmonary arterial hypertension (PAH) is a specific subtype of PH distinguished by pulmonary vascular resistance (PVR) above 2 wood units (WU) (Ruopp and Cockrill, 2022; Humbert et al., 2023; Maron, 2023). PAH patients present with significant vasoconstriction, thickening of the vessel walls, and aberrant remodeling of the vasculature (Yu et al., 2006; Humbert et al., 2019; Ruopp and Cockrill, 2022). Hypertrophy and proliferation of smooth muscle cells surrounding the vessels also contribute to elevated pulmonary pressure (Pathology and pathobiology of pulmonary hypertension: state of the art and research perspectives, 2019; Ruopp and Cockrill, 2022). Similarly, cardiomyocytes, particularly in the right ventricle that pushes blood into the lungs, exhibit hypertrophy in PAH as the heart compensates for the increased vascular resistance (Hsu et al., 2016; Pathology and pathobiology of pulmonary hypertension: state of the art and research perspectives, 2019). The increased workload and right ventricular systolic pressure (RVSP) contribute to right heart failure which is often how PAH results in death (Jacobs et al., 2014; Hsu et al., 2016).

Global prevalence of PAH is just over 2 per 100, 000 people, and there were 22, 000 deaths in the US associated with PAH in the year 2021 (Leary et al., 2025). While the overall prevalence is relatively low, the high mortality rate for those diagnosed with PAH, approximately 40% within 5 years of diagnosis (Gall et al., 2017; Ruopp and Cockrill, 2022; Dignam et al., 2024), indicates there is an urgent need for improved therapeutic strategies. Most treatments for PAH attempt to reduce pulmonary pressure by either activating vasodilative pathways such as eNOS or inhibiting vasoconstrictive pathways like endothelin (Nikkho et al., 2020; Ruopp and Cockrill, 2022; Weatherald et al., 2022; 2022 ESC/ERS guidelines for the diagnosis and treatment of pulmonary hypertension, 2023). While such treatments have increased overall survival rates of PAH patients, they do not work for all patients and their efficacy is limited (Nikkho et al., 2020; Ruopp and Cockrill, 2022; Weatherald et al., 2022; 2022 ESC/ERS guidelines for the diagnosis and treatment of pulmonary hypertension, 2023; Leary et al., 2025). Due to this, the underlying genetic mechanisms driving pathophysiology of the heart and lungs must be fully elucidated to identify superior targets for therapeutic intervention.

Studies on heritable PAH have implicated BMP, TGF-β, and Caveolin-1 (*Cav1*) as pathways where mutations increase PAH risk (Pathology and pathobiology of pulmonary hypertension: state of the art and research perspectives, 2019; Southgate et al., 2020). PAH often presents as a heritable disease linked to loss-of-function mutations in particular genes such as *Bmpr2, Cav1, Gdf2* (BMP-9), and *Smad1 (*Ma and Chung, 2014; Pathology and pathobiology of pulmonary hypertension: state of the art and research perspectives, 2019; Southgate et al., 2020)*. Bmpr2* mutations comprise the largest portion of heritable PAH patients, marking the BMP signaling pathway (and the interdependent TGF-β pathway) as targets for consideration (Southgate et al., 2020; Li and Quigley, 2024). Interestingly, the caveolae structural and scaffolding protein caveolin-1 is a PAH risk factor that is depleted in PAH patient lungs (Achcar et al., 2006; Southgate et al., 2020; Tomita et al., 2024). Considering caveolin-1 has been shown to interact with BMP and TGF receptors and modulate their signaling (Razani et al., 2001; Nohe et al., 2005) and the many roles of Cav1-regulated signaling plays in maintaining pulmonary function (Drab et al., 2001; Gairhe et al., 2021), investigations into the Cav1-BMP-TGF signaling axis would be a logical next step for improving our understanding PAH development.

Another approach which may elucidate the mechanisms of PAH development is investigating the disease’s paradoxical sex dynamic (Jacobs et al., 2014; Dignam et al., 2024). The “sex paradox” of PAH refers to how females comprise a larger proportion of PAH patients (around 63%), but male patients demonstrate approximately 10% higher mortality rates 5 years after diagnosis (Jacobs et al., 2014; Kozu et al., 2018; DesJardin et al., 2024; Dignam et al., 2024). This trend remains even when data is standardized to account for differences in income, drug use, smoking, and other factors, indicating sex itself as a risk factor (Kozu et al., 2018; DesJardin et al., 2024). However, like the other underlying mechanisms of PAH development, this dynamic is poorly understood; how sex, genotype, and hypoxia influence the known PAH-related pathways has not been fully elucidated.

## Results

### Caveolin-1 expression in the endothelium is sufficient to prevent hypoxia-induced mortality in Caveolin-1 knockout mice

**Figure 1:**
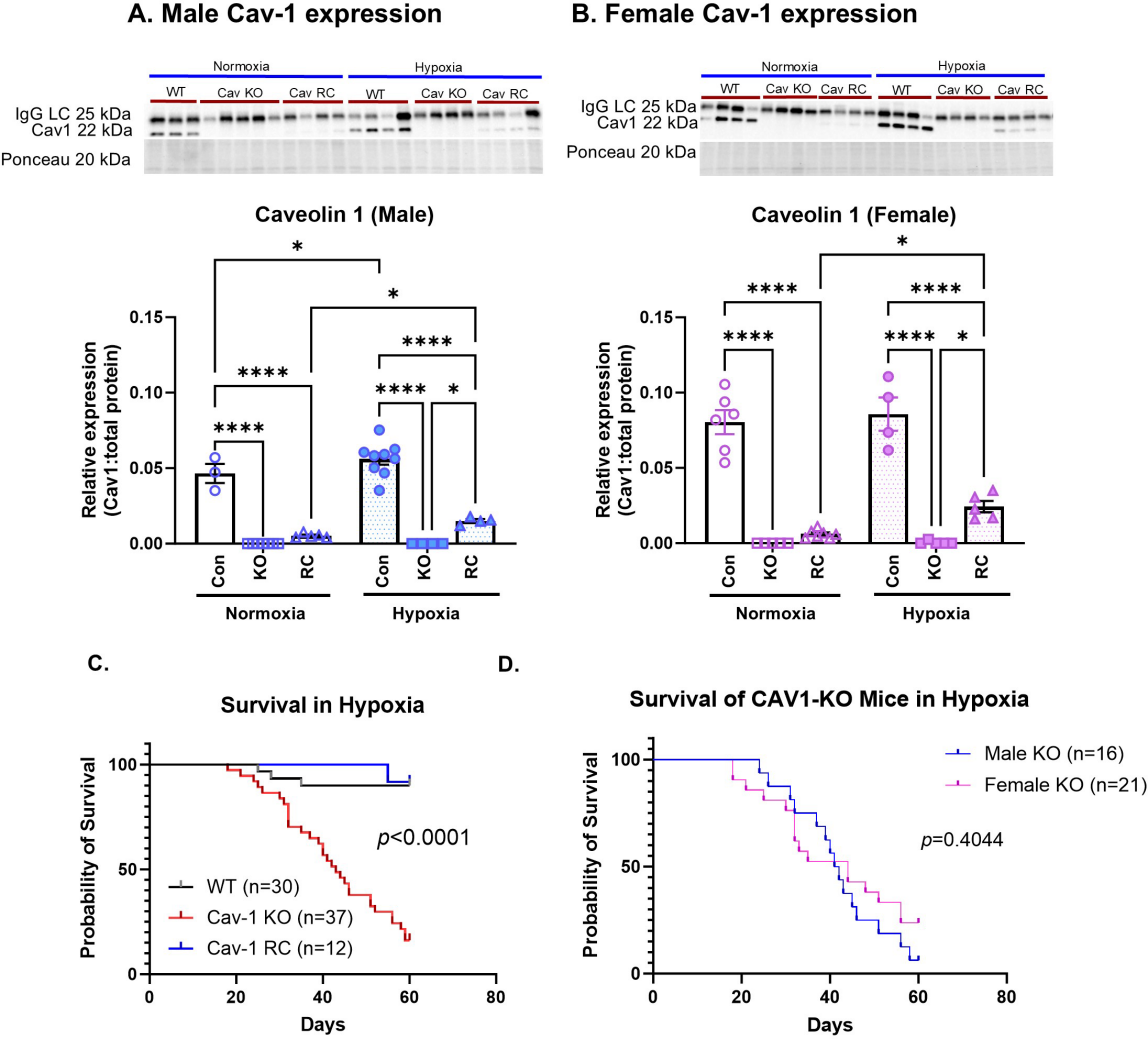
Effect of Caveolin-1 (Cav1) knockout and reconstitution on mouse survival in hypoxia. Representative Western Blots against CAV1 in **(A)** male (n = 3 – 9) and **(B)** female (n=4-7) mice after 6 weeks of hypoxia. All western blot images used for quantification are available as Source Data. Data is quantified relative to whole protein using ponceau staining. *Post-hoc* 2-way analysis of variance (ANOVA) with Bonferonni’s multiple comparisons test where *P-value < 0.05, ****P-value < 0.0001. Data presented as mean ± standard error of the mean (SEM). **(C)** Survival curves of wildtype (n=30), Caveolin-1-knockout (n=37), and reconstituted mice (n=12) in hypoxia (10% oxygen) over 60 days. **(D)** Survival curve comparing male (n=16) and female Caveolin-1 (n=21) knockout over 60 days of hypoxia. *Post-hoc* survival curve analysis was performed with Mantel-Cox comparisons.

In order to interrogate the role of endothelial caveolin-1 in the development of PAH, we utilized a *caveolin-1* global knockout model (Cav1-KO) and an EC-specific reconstitution model that retains the knockout of *caveolin-1* in non-endothelial cell types (Cav1-RC) (Murata et al., 2007) and compared these to wildtype B6/129SJ2 as background controls. Whole lungs were collected from male and female mice from all three groups for protein isolation to determine their relative levels of caveolin-1 expression (**Figures 1A, B**). As expected, caveolin-1 protein expression was nearly undetectable in both male and female Cav1-KO mice. Cav1-RC mice demonstrated low but detectable levels of expression, due to only being expressed in the endothelium. Notably, chronic hypoxia exposure induced a subtle but significant increase in caveolin-1 expression in both male and female Cav1-RC mice, and in WT male mice. This data is suggestive of potential differences between males and females in how caveolin-1 expression changes in response to hypoxia. Additionally, both WT and Cav1-RC mice demonstrated hypoxia-induced upregulation of caveolin 1, suggesting endothelial expression of caveolin 1 as playing a role in this observation.

To determine how caveolin-1 expression impacts the mouse’s ability to survive chronic hypoxia, mice of both sexes from all three groups were placed in 10% oxygen conditions using a hypoxia chamber and observed over the course of 8 weeks (**Figure 1C**). The experiment was taken to 8 weeks in order to investigate the impact of caveolin 1 expression on mortality in chronic hypoxia, as at that point the survival difference between groups was the most prominent before the death of all mice in any group. Wildtype mice experienced low rates of mortality in hypoxia with only about 10% mortality by the end of the experiment. Conversely, global Cav1-KO mice began dying after 20 days of hypoxia and ended the experiment with an approximate 89.2% mortality rate. Notably, the Cav1-RC mice survival rate in hypoxia was close to WT controls despite only expressing caveolin-1 in the endothelium. This demonstrates the key role for endothelial caveolin-1 in lung homeostasis and PAH development.

To investigate how hypoxia-induced mortality may differ by sex, the hypoxia survival experiment was conducted with male and female Cav1-KO mice over the course of 8 weeks (**Figure 1D**). While the overall survival rate was not significantly different, an interesting pattern emerged that may mirror the sex dynamics seen in human patients. In our study, we found that female mice began dying earlier than males at about 20 days of hypoxia. However, by the end of the study, a larger number of females had survived compared to males (approximately 23.8% vs. 8.3%), which mirrors the broad trends seen in human PAH patients where females develop PAH at higher rates but also experience lower PAH mortality rates.

### Cav1-KO mice in chronic hypoxia possess pathophysiology of severe PAH

**Figure 2:**
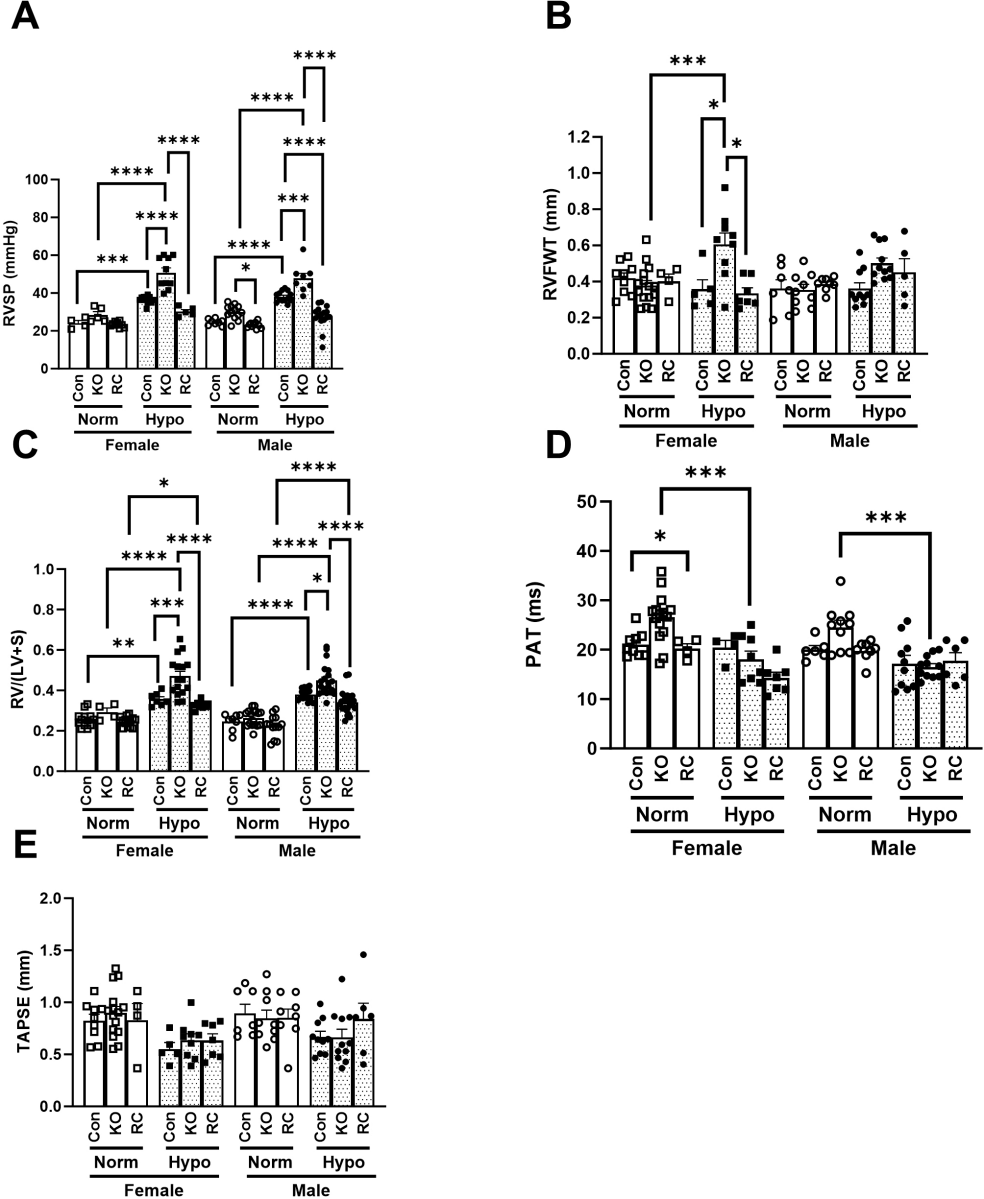
Right ventricular (RV) function and structure of Caveolin-1 (Cav1) global knockout or Caveolin-1 endothelial-specific reconstituted male and female mice exposed to 6 weeks of hypoxia. Control (Con), Cav1 global knockout (KO), and Caveolin-1 endothelial-specific reconstituted (RC) are plotted by normoxia (Normoxia), white bars, or hypoxia (Hypo), dotted bars. **(A)** Right ventricular systolic pressure (RVSP) (n = 4 – 16) **(B)** Right ventricular free wall thickness (RVFWT) (n = 4 – 11) **(C)** Right Ventricular Hypertrophy (RVH) as determined by the weight ratio of the right ventricle divided by the sum of left ventricle and septum (RV/(LV+S)) (n = 4 – 25) **(D)** Pulmonary acceleration time (PAT) (n = 4 – 15) **(E)** Tricuspid annular plane systolic excursion (TAPSE) (n = 4 – 15). *Post-hoc* 3-way ANOVA analysis with Bonferonni’s multiple comparisons tests where *P-value < 0.05, **P-value < 0.01, ***P-value <0.001, ****P-value < 0.0001. Data presented as mean ± standard error of the mean (SEM).

To assess the pulmonary and cardiac physiology of mice in hypoxic conditions that lead to increased mortality, both sexes and the three genetic groups underwent chronic hypoxia exposure for 6 weeks and were compared to normoxic controls using echocardiography and hemodynamic measurements. Measurements of right ventricular systolic pressure (RVSP) were taken as an approximate measurement of pulmonary hypertension in the mouse (**Figure 2A**). For both males and females, hypoxia alone significantly increased RVSP compared to normoxic controls. Notably, Cav1-KO males demonstrated significantly higher pressure than their wildtype counterparts in normoxia, while Cav1-KO females did not demonstrate a significantly higher normoxic RVSP, hinting at higher male susceptibility to genetic PAH risk factors. Notably, the restoration of endothelial Cav1 expression (Cav1-RC) restored RVSP to wildtype levels, highlighting the key role of endothelial Cav1 in pulmonary vascular homeostasis. Right ventricular free wall thickness (RVFWT) measurements demonstrate the advantage of combining hypoxia with c*aveolin-1* knockout for modeling disease pathology (**Figure 2B**). For females, Cav1-KO alone and hypoxia alone were insufficient to significantly increase RVFWT, but hypoxic Cav1-KO mice demonstrated significantly higher RVFWT compared to normoxic controls. Male Cav1-KO mice in hypoxia demonstrated a similar but subtle trend that was not statistically significant compared to the male wildtype and normoxia controls, hinting at sex differences in PAH pathophysiology. Right ventricular hypertrophy (RVH) was measured directly as RV/(LV+S) (**Figure 2C**), known as the Fulton Index. RVH followed a similar pattern to RVSP where hypoxia alone is sufficient to induce a significant increase across both sexes, but the largest increase in right ventricular hypertrophy was seen when combining hypoxia and CAV1-KO. Similar to previous experiments, Cav1-RC rescued right ventricular hypertrophy to wildtype levels. Pulmonary acceleration time (PAT) was also measured and found to be increased in Cav1-KO, particularly in females, while hypoxia lowered the measurement in females (**Figure 2D**). Tricuspid annular plane systolic excursion (TAPSE) measurements (**Figure 2E**) showed minor changes in our experiment, with three-way ANOVA identifying hypoxia as a significant factor (*p* = 0.0002) but not gender or genotype. Overall, for both males and females the combination of hypoxia with Cav1-KO produced the most severe form of PAH as shown by elevated RVSP, RVH, and RVFWT, but there were also subtle differences in the pathophysiology observed in male and female mice. Interestingly, while normoxic Cav1-KO males demonstrate a slightly elevated RVSP, they do not also possess elevated RVH, RVFWT, or PAT. This could suggest a threshold of RVSP pressure required to develop the subsequent pathophysiology of hypertrophy and ventricular remodeling seen in PAH. Based on the combination of high mortality rates and elevated RVSP, RVFWT, and RVH measurements, we will refer to the hypoxic Cav1-KO mice as having pulmonary arterial hypertension (PAH) moving forward (Arnold and Chen, 2009).

### Cardiac echocardiography demonstrates sex differences in cardiac physiology in pulmonary arterial hypertension

**Figure 3:**
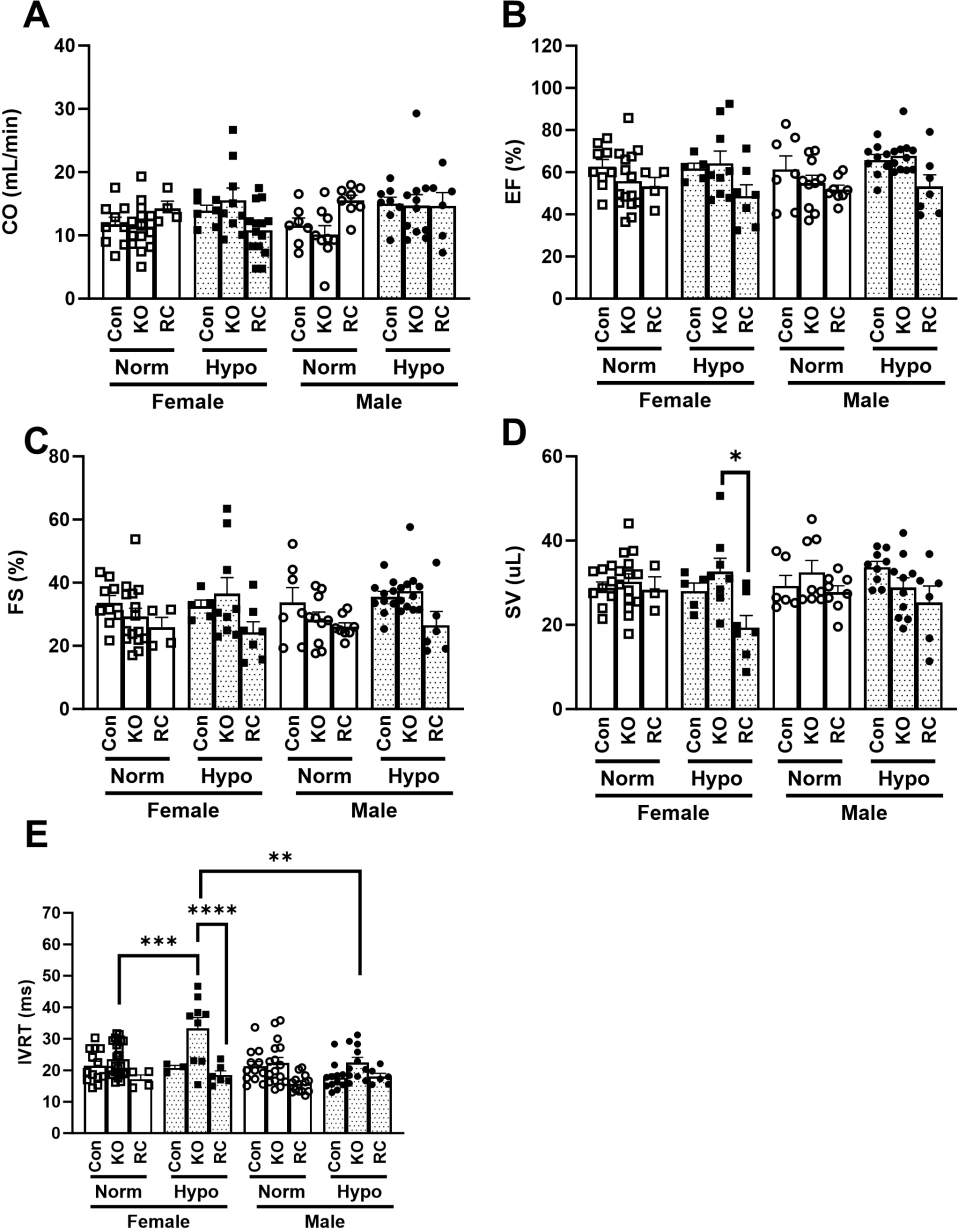
Cardiac echocardiography of Caveolin-1 (Cav1) global knockout or Caveolin-1 endothelial-specific reconstituted male and female mice exposed to 6 weeks of hypoxia. Control (Con), Cav1 global knockout (KO), and Caveolin-1 endothelial-specific reconstituted (RC) are plotted by normoxia (Normoxia), white bars, or hypoxia (Hypo), dotted bars. **(A)** Cardiac Output (CO) (n = 4 – 15) **(B)** Ejection Fraction (EF) (n = 4 – 15) **C)** Fractional Shortening (FS) (n = 4 – 15) **(D)** Stroke Volume (SV) (n = 4 – 15) **(E)** Isovolumetric relaxation time (IVRT) (n = 4 – 25). *Post-hoc* 3-way ANOVA analysis with Bonferonni’s multiple comparisons test where *P-value < 0.05, **P-value < 0.01, ***P-value < 0.001, ****P-value < 0.0001. Data presented as mean ± standard error of the mean (SEM).

Cardiac echocardiography measurements were used to determine the effect of hypoxia and Cav1 expression on cardiac function. Cardiac output (CO) did not show consistent changes across groups, although three-way ANOVA found hypoxia status to be a significant factor in our CO data (*p* = 0.0449) and further significant in conjunction with genotype (p=0.0060) (**Figure 3A**). Ejection fraction (EF) measurements (**Figure 3B**) likewise did not show consistent trends by multiple comparison analysis, though genotype was found to be a significant factor (*p* = 0.0030). This same trend continues with the fractional shortening (FS) measurements (**Figure 3C**) where there was no significance in multiple comparisons but genotype was found to be a significant factor (*p* = 0.0029). Stroke volume (SV) (**Figure 3D**) was mostly unchanged in our experiment except for a surprising decrease in SV for Cav1-RC females in hypoxia, possibly due to loss of Cav1 in pulmonary SMCs and cardiomyocytes. Finally, isovolumetric relaxation time (IVRT) showed the most interesting trend in our cardiac data (**Figure 3E**), where IVRT is substantially increased in female Cav1-KO mice in hypoxia and recovered by Cav1-RC, while no such trend was seen in males except for a slight decrease in normoxic Cav1-RC IVRT. Additionally, there is a significant difference between male and female IVRT when comparing Cav1-KO hypoxic mice, with female IVRT being significantly higher. Overall, we see that the effect of PAH on cardiac function shows differences based on sex that may provide a hint at the underlying compensatory mechanisms that account for differences in patient outcomes.

### Blood gas measurements reveal sex differences in oxygen saturation in hypoxic Cav1-KO mice

**Figure 4:**
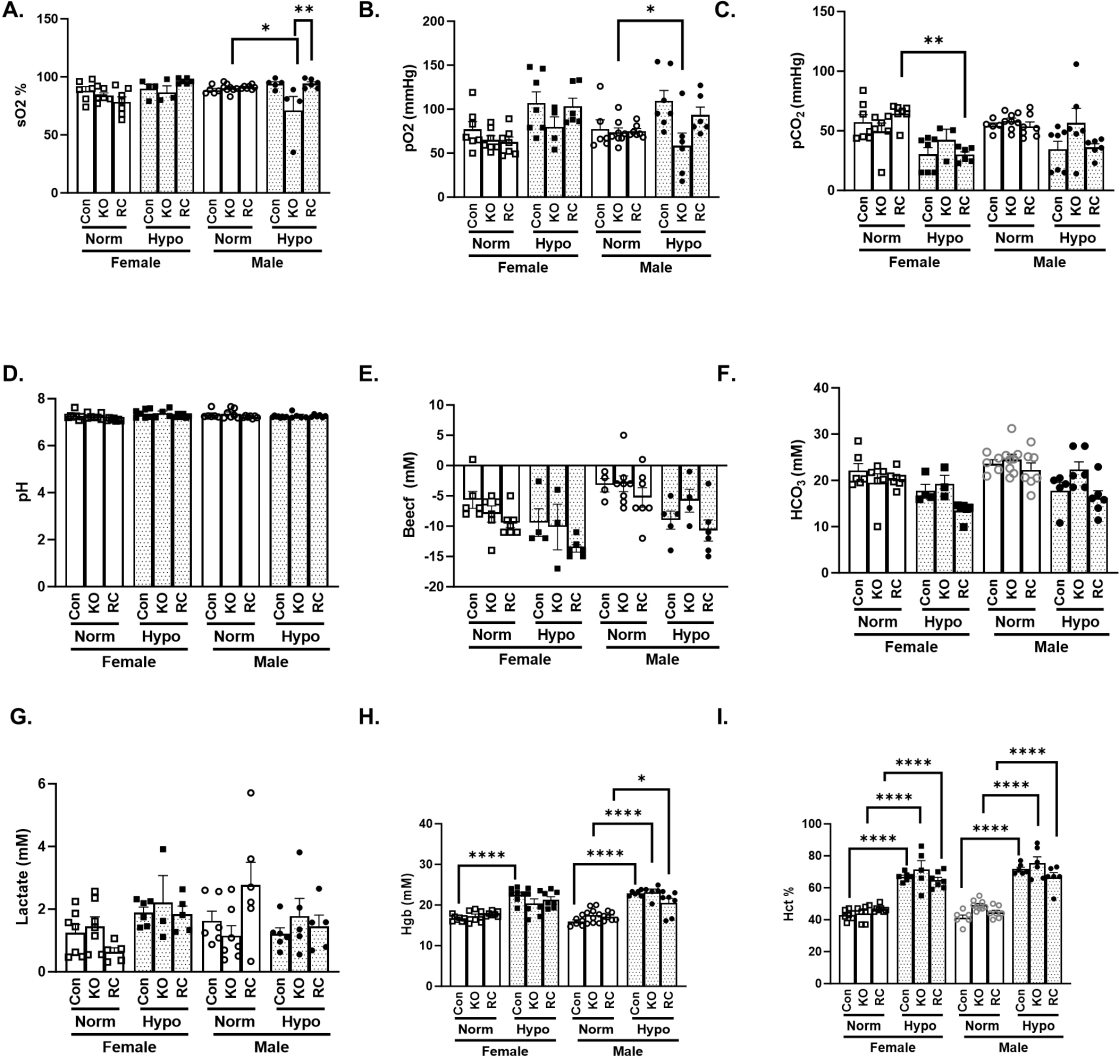
Blood gas measurements of Caveolin-1 (Cav1) global knockout or Caveolin-1 endothelial-specific reconstituted male and female mice exposed to 6 weeks of hypoxia. Control (Con), Cav1 global knockout (KO), and Caveolin-1 endothelial-specific reconstituted (RC) are plotted by normoxia (Normoxia, white bars), or hypoxia (Hypo, dotted bars). **(A)** Oxygen saturation (sO2) (n = 3 – 8) **(B)** Partial pressure of oxygen (pO2) (n = 4 – 7) **(C)** Partial pressure of carbon dioxide (pCO2) (n = 3 – 8) **(D)** pH levels (pH) (n = 4 – 8) **(E)** Extracellular base excess (BeCef) (n = 3 – 8) **(F)** Bicarbonate (HCO3) (n = 3 – 8) **(G)** Lactate (n = 3 – 7) **(H)** Hemoglobin (Hgb) (n = 5 – 9) **(I)** Hemocrit (Hct) (n = 5 – 9). *Post-hoc* 3-way ANOVA analysis with Bonferonni’s multiple comparisons test where *P-value < 0.05, **P-value < 0.01, ****P-value < 0.0001. Data presented as mean ± standard error of the mean SEM).

To determine how hypoxia and Cav-1 expression alter the levels of blood gases and respiratory metabolites, blood was collected and analyzed after 6 weeks of normoxia or hypoxia (**Figure 4**). Measurement of oxygen saturation (**Figure 4A**) shows the most distinct sex pattern, with males demonstrating a significant decrease in oxygen saturation in hypoxic Cav1-KO compared to normoxic and wildtype controls. Additionally, the reduction in oxygen saturation was rescued by EC-specific Cav1 reconstitution (Cav1-RC). Measurement of the partial pressure of oxygen (pO_2_) (**Figure 4B**) showed a similar trend where both males and females experience an increase in oxygen pressure during chronic hypoxia, but only males had a statistically significant loss of pO_2_ in hypoxic Cav1-KO compared to normoxic Cav1-KO. In fact, male Cav1-KO in hypoxia demonstrated a pO_2_ even lower than the male normoxia samples, demonstrating a male-specific change in blood oxygen pressure. Genotype (*p=*0.0032) and hypoxia (*p=*0.005) were both significant factors for oxygen pressure variation. Partial pressure of carbon dioxide (pCO_2_) measurements (**Figure 4C**) showed how both males and females experienced a decrease in pCO_2_ in hypoxic conditions, mirroring the pattern seen with pO_2_ in reverse. Notably, the increase in pCO_2_ in Cav1-KO exposed to hypoxia was significant in males but not in females. Taken together, this indicates that Cav1-KO hypoxic males experience a more notable impact to their blood oxygen and carbon dioxide levels compared to females, which may have contributed to reduced survival. Blood pH levels did not change across any of the groups (**Figure 4D**). Despite this, there were subtle changes to extracellular base excess (BEECF) levels (**Figure 4E**), primarily a subtle decrease for both males and females in hypoxia. While multiple comparisons did not show significance between groups differing by only factor, sex (*p=*0.0010), hypoxia (*p<*0.0001), and genotype (*p* = 0.0081) were all found to be significant factors in BEECF variance in this experiment. Similarly, blood bicarbonate levels did not show differences in multiple comparisons but sex (*p=*0.0072), hypoxia (*p<*0.0001), and genotype (*p=*0.0055) were significant factors (**Figure 4F**). Lactate measurements (**Figure 4G**) showed an interesting trend where generally female lactate levels increased in hypoxic conditions, indicating a greater reliance on glycolysis and nonaerobic respiration. The pattern was far less consistent in males, with the highest levels being seen in normoxic Cav1-RC males. While specific comparisons did not find significance, the combination of sex and hypoxia was found to be a significant factor in blood lactate levels (*p=*0.0086). This could hint at a compensatory mechanism in female respiration, where glycolysis makes up for reduction in aerobic respiration in hypoxia that was not seen in male mice. Hemoglobin levels (**Figure 4H**) did not show a strong sex-specific pattern, but there is a consistent trend where hypoxia groups showed a significant increase in hemoglobin levels compared to normoxic controls, likely to compensate for low oxygen levels, particularly in males. Finally, hematocrit (Hct), which represents the percentage of blood volume that is made up of red blood cells (**Figure 4I**), was significantly increased in males and females exposed to hypoxia. Taken together, this data indicates there are sex-specific differences in how blood oxygen and lactate levels change in hypoxic conditions. However, across all the physiological data, only IVRT demonstrated a significant difference in Cav1-KO hypoxia mice when males and females were directly compared. While notable differences within male and female groups were found, this indicated physiological markers alone could not account for the difference in hypoxia survivability and PAH development. Thus, we pursued RNA-sequencing of the lungs in order to gather more information on the underlying transcriptomics.

### RNA-sequencing of PAH mouse lungs implicates Caveolin-1 as a key regulator of gene expression important for cilia formation and muscle development

Control, Cav1-KO, and Cav1-RC mice underwent 6 weeks exposure to normoxic or hypoxic conditions before lungs were isolated for RNA sequencing. A series of pairwise comparisons were performed for differential gene expression (DEG) analysis, with male and female samples separated. The total number of up- and down-regulated DEGs are shown for each notable pairwise comparison in **Supplementary Figure 1**. Principle component analysis (PCA) was performed to determine intra-group consistency (**Supplementary Figure 2**). The DEG analysis was also used for gene ontology (GO) analysis, which categorizes genes by biological process. qPCR validation against notable DEGs is shown in **Supplementary Figure 3**. Here we compared RT-qPCR using RNA from comparable experimental mice against genes from notable pathways implicated in PAH: *DKK2, NOS3 (*eNOS), *RBPJ*, and BMPR2. These values were compared to fragments per kilobase of transcript per million mapped reads (FPKM) values for the same genes from the RNA-sequencing data. Here, the trends seen in gene expression changes can be seen in both datasets, particularly the Cav1-KO induced upregulation of *DKK2* and *eNOS*. *BMPR2* does not show particularly strong trends in either reading and *RBPJ* qPCR shows a weaker trend than its FPKM counterpart, but this may be attributable to the increased variance in RT-qPCR data.

**Figure 5:**
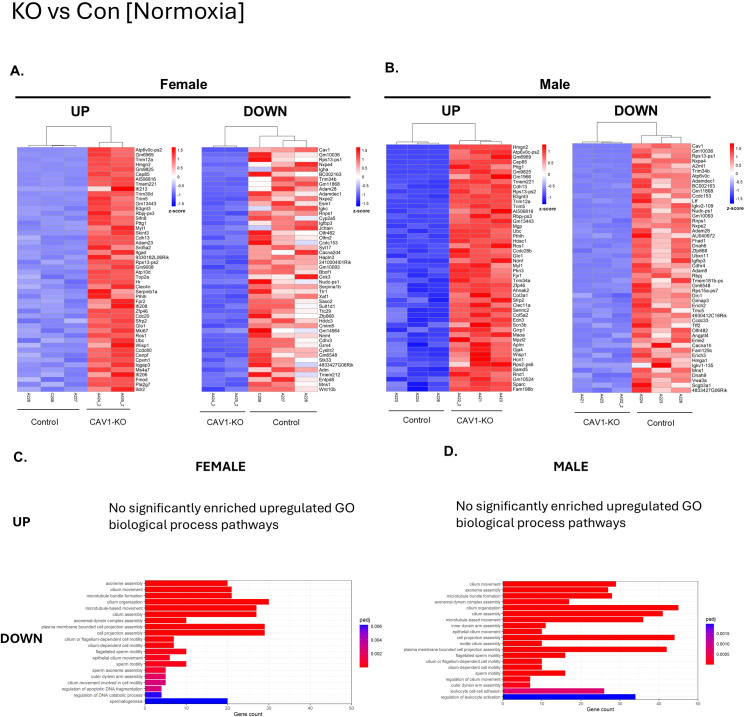
Pairwise comparison of differentially expressed genes between Caveolin-1 knockout (Cav1-KO) and wildtype control (Control) mouse lungs in normoxic conditions. Male (control n=3, Cav1-KO n=3) and female (control n=3, Cav1-KO n=2) comparisons were performed separately. The top 50 differentially expressed genes (DEG) upregulated and downregulated are shown in descending order of significance based on adjusted P-value (P_adj_) for **(A)** female and **(B)** male lungs. DEG thresholds were set at Log2Foldchange > 1, P_adj_ <0.05. Z-score coloring represents relative gene expression across all groups. Samples are sorted by complete hierarchical clustering. Gene Ontology (GO) analysis for **(C)** Female and **(D)** Male samples are shown as the top 20 gene ontologies in descending order of P_adj_. The X-axis represents the number of DEGs in this comparison that underlies the significant GO.

Comparing Cav1-KO to wildtype controls in normoxic conditions (**Figure 5**) revealed a major downregulation of genes belonging to GOs related to cilia such as cilium movement, cilium organization, and cilium assembly. Particular downregulated genes across both sexes include structural protein coding gene *MNS1* and transcription factor *FOXJ1*, hinting at dysfunction of either ciliated cells (Yu et al., 2008) and/or ciliated endothelial cells in Cav1-KO mouse lungs. Conversely, comparing hypoxic Cav1-KO to normoxic Cav1-KO (**Supplementary Figure 4**) suggests chronic hypoxia may upregulate these same GOs. While the most significant GOs for both males and females were related to cilia and cell motility, males also demonstrated significantly downregulated gene expression in GOs such as leukocyte cell-cell adhesion, regulation of leukocyte activation, and T and B cell activation that suggests a male-specific dysregulation of immune activation in Cav1-KO mice.

### Knockout of Cav-1 in males upregulates muscle cell development genes, while knockout of Cav-1 in females downregulates BMP signaling and angiogenesis

**Figure 6:**
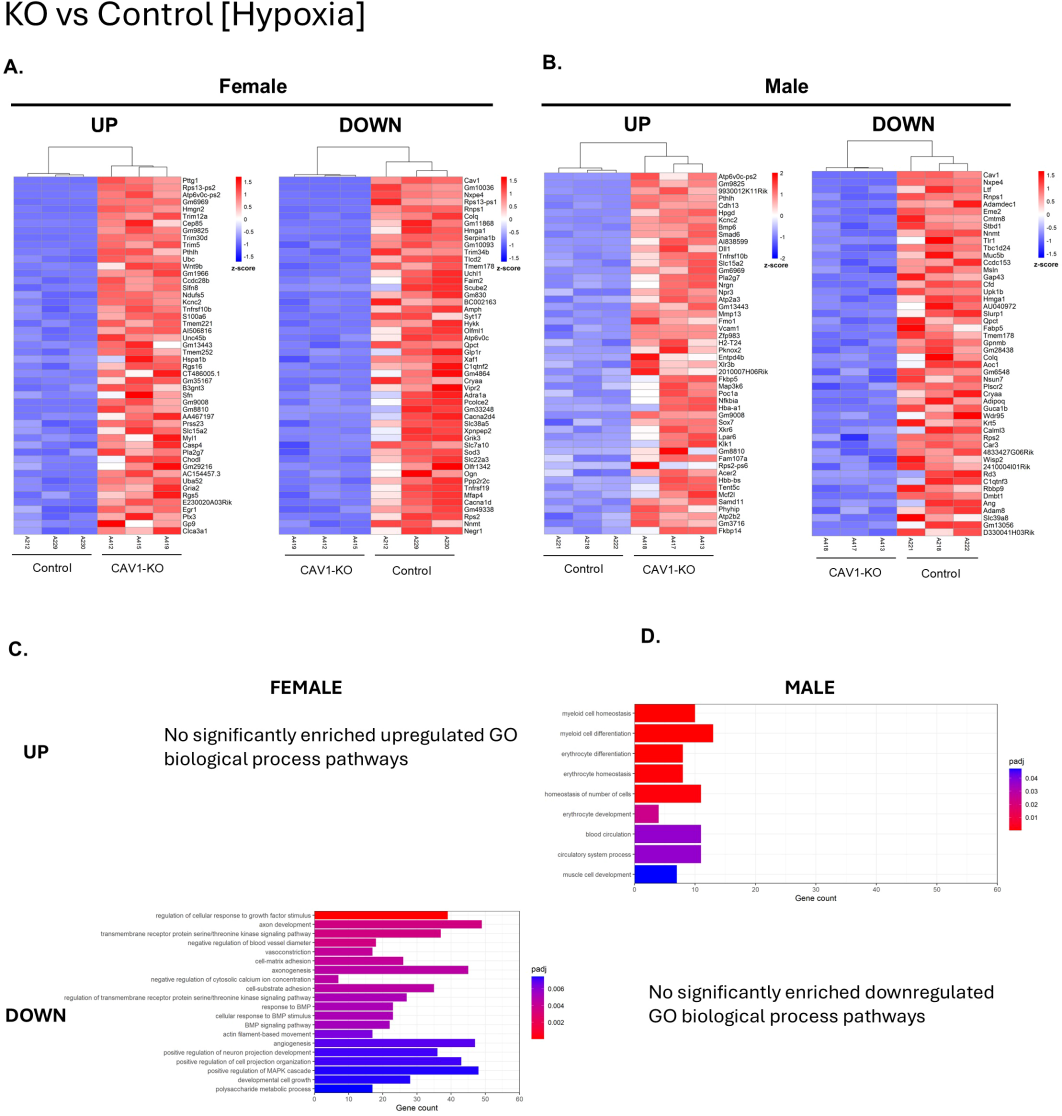
Pairwise comparison of differentially expressed genes between Caveolin-1 knockout (Cav1-KO) and wildtype control (Control) mouse lungs in hypoxic conditions. Male and female comparisons were performed separately (n=3 in each group). The top 50 differentially expressed genes (DEG) upregulated and downregulated are shown in descending order of significance based on adjusted P-value (P_adj_) for **(A)** female and **(B)** male lungs. DEG thresholds were set at Log2Foldchange > 1, P_adj_ <0.05. Z-score coloring represents relative gene expression across all groups. Samples are sorted by complete hierarchical clustering. Gene Ontology (GO) analysis for **(C)** Female and **(D)** Male samples are shown as the top 20 gene ontologies in descending order of P_adj_. The X-axis represents the number of DEGs in this comparison that underlies the significant GO.

Comparing Cav1-KO to wildtype controls in the context of hypoxia (**Figure 6**) demonstrates even more sex differences in gene expression. Notably, while female Cav1-KO hypoxic mice had 491 upregulated DEGs, no biological process GOs were significant for this comparison. This may be because many of the genes were related to cell compartment GOs, such as those associated with spindle formation and kinetochore. These GOs were not significant in males, and instead there was an upregulation of pathways related to myeloid cell and erythrocyte development. Another interesting finding was the upregulation of genes in the muscle cell development GO only in males. In Cav1-KO, this is only seen in hypoxic males, but notably many muscle development related pathways are found to be downregulated when comparing hypoxic mice to normoxic mice (**Supplementary Figures 3**, **4**) in both males and females. However, females notably demonstrated fewer DEGs related to these pathways in normoxia. This could indicate sex differences in the transcriptomics that drive smooth muscle hypertrophy in male PAH compared to females. Conversely, hypoxic Cav1-KO males did not have any significantly downregulated biological process GOs compared to hypoxic WT controls, whereas females demonstrated several downregulated pathways. BMP signaling pathway, response to BMP, and cellular response to BMP stimulus were all downregulated in female hypoxic Cav1-KO compared to hypoxic control. Though males did not demonstrate significant BMP-related GOs, *Bmp6* and *Smad6* were both upregulated in males, which were not DEGs in the same female comparison. This suggests males and females may regulate BMP signaling in sex-specific patterns in response to caveolin-1 depletion and hypoxia. Vascular GOs such as vasoconstriction and angiogenesis were downregulated in females, possibly as a compensatory mechanism. Taken together, this suggests that BMP signaling, angiogenesis, and muscle cell development are transcriptionally regulated in a sex-specific manner that may account for differences in PAH outcome.

### Reconstitution of endothelial caveolin-1 increases gene expression of cilia-related pathways, muscle cell development, and immune cell response

**Figure 7:**
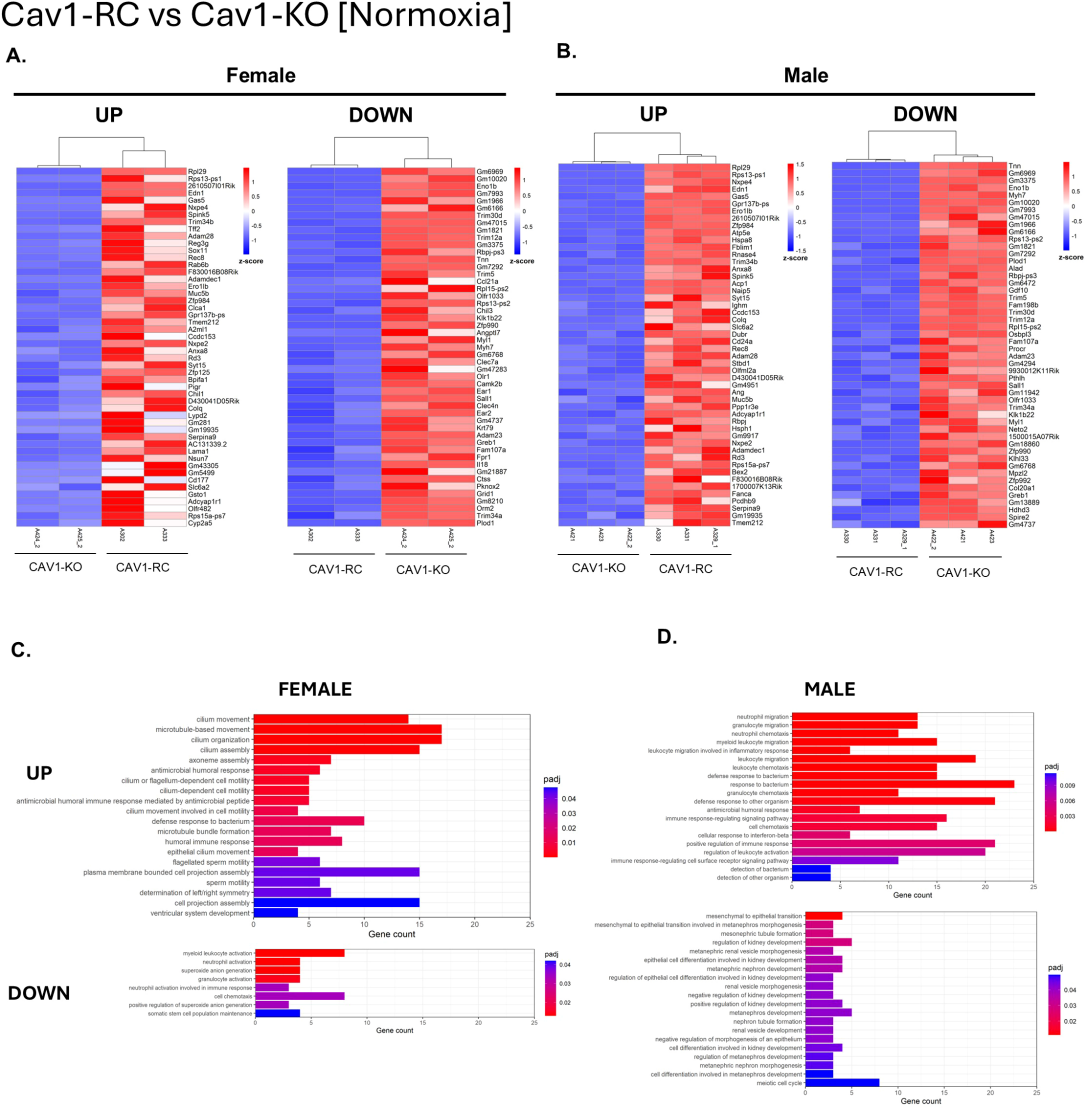
Pairwise comparison of differentially expressed genes between endothelial Caveolin-1 reconsituted (Cav1-RC) and Caveolin-1 knockout (Cav1-KO) mouse lungs in normoxic conditions. Male (Cav1-KO n=3, Cav1-RC n=3) and female (Cav1-KO n=2, Cav1-RC n=3) comparisons were performed separately. The top 50 differentially expressed genes (DEG) upregulated and downregulated are shown in descending order of significance based on adjusted P-value (P_adj_) for **(A)** female and **(B)** male lungs. DEG thresholds were set at Log2Foldchange > 1, P_adj_ <0.05. Z-score coloring represents relative gene expression across all groups. Samples are sorted by complete hierarchical clustering. Gene Ontology (GO) analysis for **(C)** Female and **(D)** Male samples are shown as the top 20 gene ontologies in descending order of P_adj_. The X-axis represents the number of DEGs in this comparison that underlies the significant GO.

**Figure 8:**
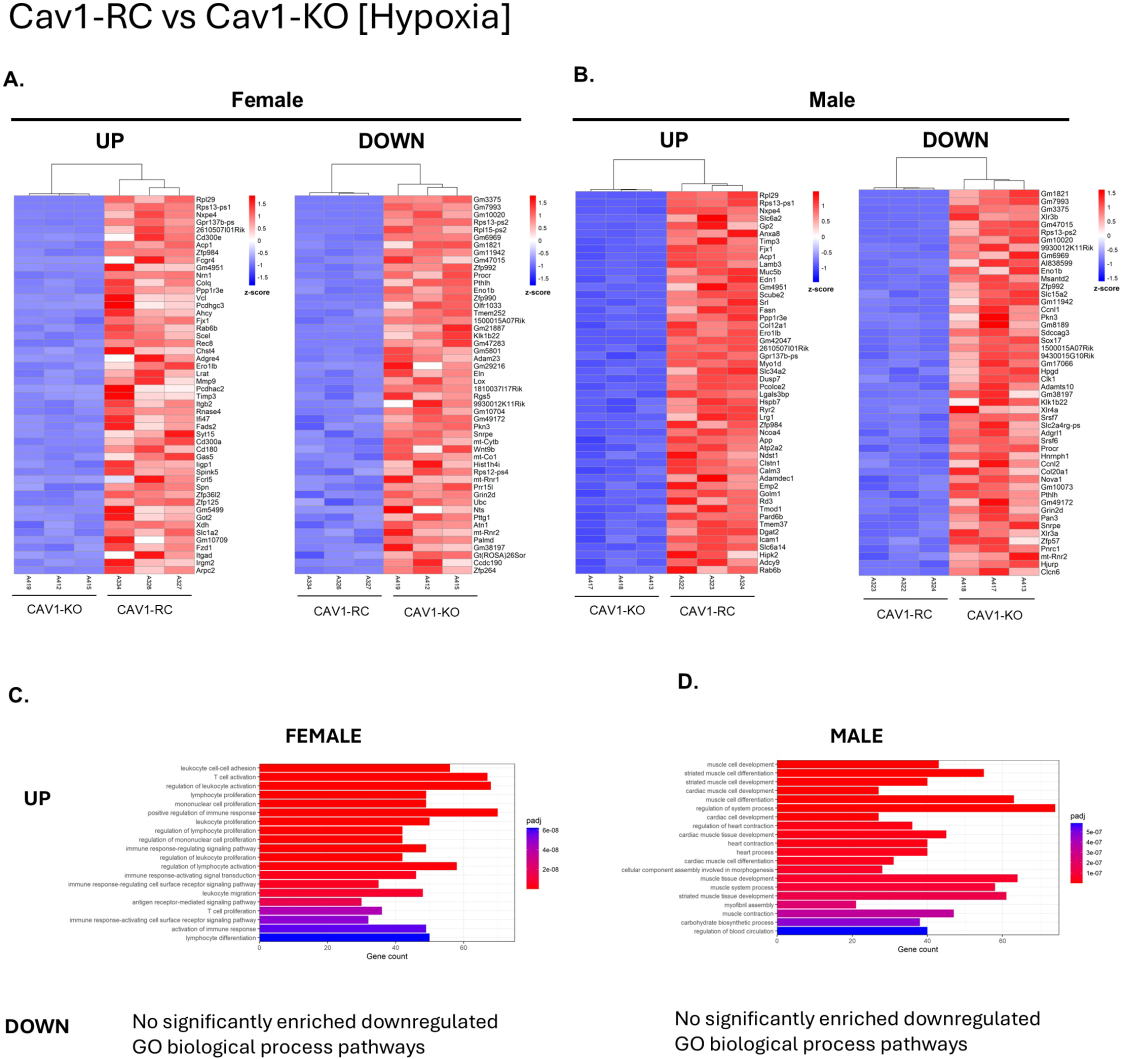
Pairwise comparison of differentially expressed genes endothelial Caveolin-1 reconsituted (Cav1-RC) and Caveolin-1 knockout (Cav1-KO) mouse lungs in hypoxic conditions. Male and female comparisons were performed separately (n=3 for each group). The top 50 differentially expressed genes (DEG) upregulated and downregulated are shown in descending order of significance based on adjusted P-value (P_adj_) for **(A)** female and **(B)** male lungs. DEG thresholds were set at Log2Foldchange > 1, P_adj_ <0.05. Z-score coloring represents relative gene expression across all groups. Samples are sorted by complete hierarchical clustering. Gene Ontology (GO) analysis for **(C)** Female and **(D)** Male samples are shown as the top 20 gene ontologies in descending order of P_adj_. The X-axis represents the number of DEGs in this comparison that underlies the significant GO.

To investigate the specific role of endothelial caveolin-1 in PAH development, we performed a pairwise comparison between Cav1-RC mice, where caveolin-1 is only expressed in the endothelium, and Cav1-KO mice, where caveolin-1 is globally knocked out. In female Cav1-RC normoxia mice (**Figure 7**), expression of cilia-related gene ontologies discussed previously was restored, while those same GOs were not found to be upregulated in either male Cav1-RC group. Interestingly, two of the upregulated GOs in male Cav1-RC were downregulated in female Cav1-RC: myeloid leukocyte activation and cell chemotaxis. Both normoxic Cav1-KO males and females showed several upregulated neutrophil, leukocyte, and interferon-beta response pathways, though the GOs did not make the top 20 upregulated pathways in females. Additionally, male Cav1-RC mice showed an upregulation of several GOs related to muscle cell development and differentiation that were also upregulated in female Cav1-RC, though to a less significant degree. In hypoxia (**Figure 8**), comparing male Cav1-RC to Cav1-KO showed many muscle related pathways and the specific gene *myom2* in males being upregulated that were also upregulated in the normoxic comparison. Since many of these GOs were also upregulated in Cav1-KO and downregulated in hypoxia, this suggests the changes to muscle cell development gene expression may not be as simple as upregulation or downregulation, but rather that Caveolin-1 maintains intercellular signaling needed to regulate healthy muscle development that may be disrupted by chronic hypoxia. The muscle cell development GO was also upregulated in female hypoxia, though to a less significant degree. On the other hand, hypoxic female Cav1-RC mice showed upregulation of many of the same neutrophil and leukocyte related gene ontologies seen in the male normoxia comparison. This suggests that immune dysregulation and muscle development may be a common factor in both male and female PAH, but the degree of change and factors required to induce such changes show a sex-specific pattern. Interestingly, across both males and females a variety of differentiation-related gene ontologies were found to be upregulated including muscle and cardiomyocyte cell differentiation, and a female-specific upregulation of T-Cell, B-cell, lymphocyte, and myeloid differentiation. This is particularly surprising considering the Cav1-RC mice only restore Cav1 expression to the endothelium. This suggests a role for Cav1-mediated endothelial signaling in maintaining adjacent cell identities. Finally, though not in the top 20 upregulated GOs, hypoxic Cav1-RC males demonstrated a significant increase in the BMP signaling pathway GO with upregulated genes such as *BMP5* and *GDF2* (BMP9). Notably female mice also saw restored expression of BMP genes such as *RBPJ* and *BMP5* though the gene ontology itself was not significant. Thus, in our model Cav1 depletion leads to a reduction of BMP signaling that is then restored with reconstitution of Cav1 in the endothelial cells that coincides with restored survivability in hypoxia.

### Changes to Caveolin-1 expression results in changes in the expression of known PAH risk factors

**Figure 9:**
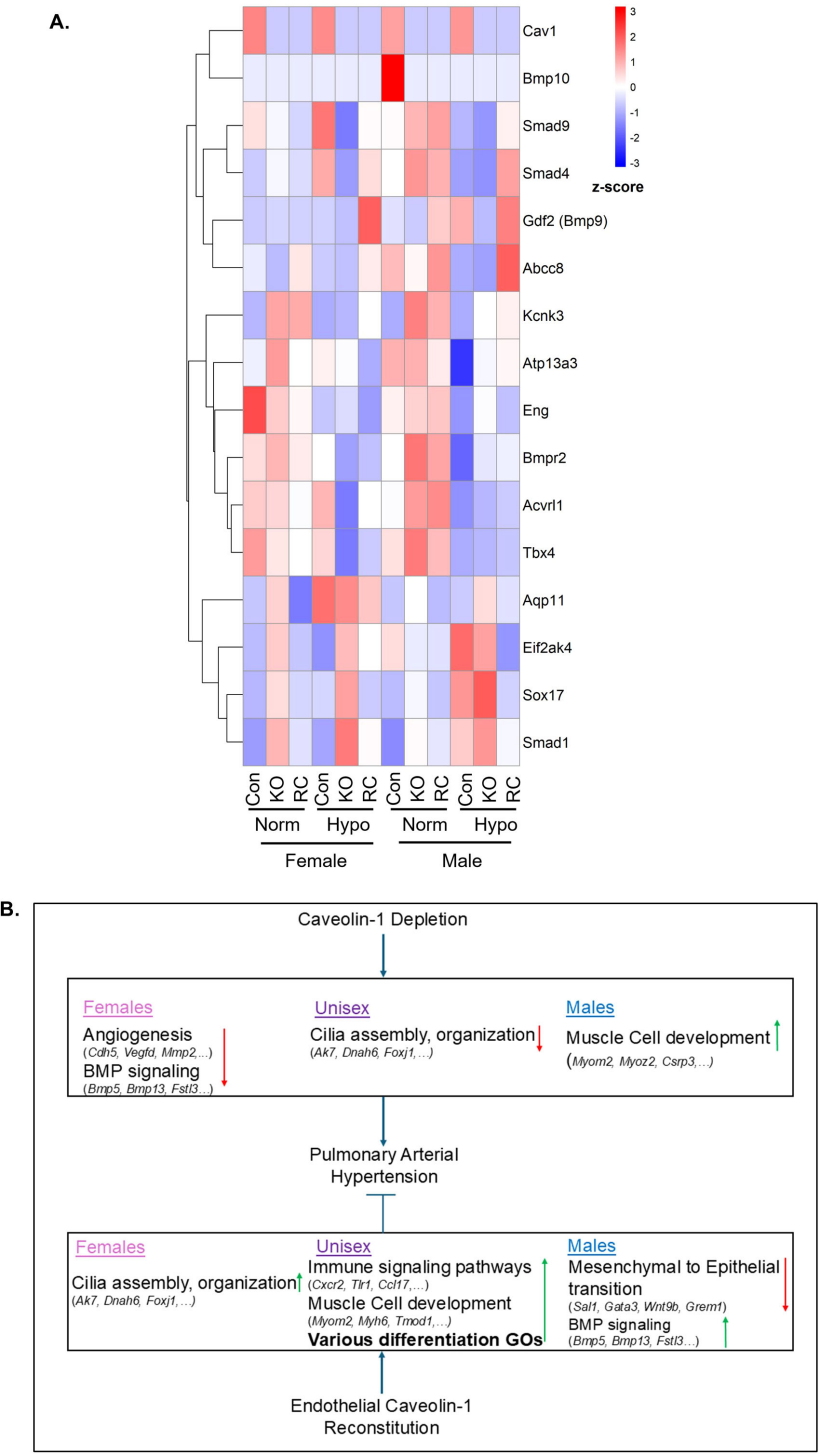
Summary of the effect of Caveolin-1 (Cav1) genetic status, hypoxia, and sex on the expression of known pulmonary arterial hypertension (PAH) risk factors and summary figure of transcriptional changes found in Cav1 genetic modulation. **(A)** A heatmap of genes associated with heritable PAH risk. Each group represents the average expression of these genes across all samples in the group. Genes are organized by hierarchical clustering to demonstrate similar patterns of expression across the groups. (n = 2 – 3) **(B)** Summary figure showing which transcriptional pathways are modulated during global Cav1 knockout or endothelial Cav1 reconstitution. Gene ontologies are distinguished by which sex, or both, they show significant change.

In order to determine how modulating *caveolin-1* expression impacts the expression of other genes associated with heritable PAH risk, we generated a heatmap of previously characterized PAH risk factor genes that shows the average expression in each experimental group (**Figure 9A**). Smad9 and Smad4 demonstrate similar expression patterns as evidenced by hierarchical clustering indicating a reduction of expression in Cav1-KO in hypoxia and hinting at some loss and/or dysregulation of BMP signaling in both males and females. Similarly, BMP-related genes such as BMPR2 are downregulated compared to their respective normoxia wildtype groups. Smad1, on the other hand, is upregulated in male and female KO hypoxia, indicating that signaling in PAH may favor TGF-β over BMP signaling. Notably many of the genes on the lower portion of the heatmap such as *Tbx4, Sox17*, and *Smad1* appear generally downregulated with Cav1-KO and then restored in Cav1-RC (or the inverse pattern with *Eif2ak4 and Aqp11*). Overall, these findings emphasize the role that endothelial caveolin-1 expression plays in the regulation of many of these genes known to confer risk for developing PAH.

## Discussion

In this study we investigated the role of caveolin-1 in PAH development by utilizing a hypoxia-induced PAH model in conjunction with models of global Cav1 knockout (Cav1-KO) and endothelial-exclusive Cav1 expression (Cav1-RC). Our data demonstrated that expressing Cav1 in the endothelium restored mouse survivability in hypoxia compared to Cav1-KO counterparts, and that males died at higher rates in a manner comparable to human PAH patients. Physiological data demonstrated that both male and female Cav1-KO mice in hypoxia experienced increased RVSP and RVH, but there was also a female-specific impact on RVFWT, which was not significantly increased in males. RNA-sequencing of these mouse lungs helped us elucidate the underlying transcriptomic changes in our model of PAH, and a summary of the transcriptomic changes are shown in **Figure 9B**. We propose that subtle sex-specific transcriptomic changes to angiogenesis, BMP signaling, immune regulation, and muscle cell differentiation in Cav1-KO and sex-specific dynamics of RVFWT, blood oxygen levels, and lactate may account for the sex differences we found in hypoxia survival which deserve further investigation. Previous literature points to connections between most of these pathways and the pathophysiology of PAH development (Southgate et al., 2020; Erewele et al., 2022), but our data specifically ties these pathways to modulation of caveolin-1 expression. One particularly surprising finding was the GOs related to cilia assembly and organization. A recent study has tied caveolin-1 expression to changes in multicilliated epithelial cell differentiation in the airways (Wickham, 2016), and there is evidence of changes to endothelial cilia development in the context of PAH (Ali, 2024). Thus, further studies are necessary to determine whether these transcriptomic changes impact either ciliated cell morphology or functioning of endothelial cilia.

The results of this project also demonstrate the value of caveolin-1 knockout mice for studying hypoxia-induced PAH. Many animal models of PAH have been utilized, including chronic hypoxia alone (Boucherat et al., 2022), and our results show that hypoxia is sufficient to increase RVSP and RV hypertrophy. However, other physiological changes consistent with PAH, particularly an increase in RVFWT, only presented in Cav1-KO mice in chronic hypoxia. Additionally, the combination of Cav1-KO and hypoxia further increased RVSP and RV hypertrophy, positioning this double-hit model as one of severe PAH. While fully elucidating the complexities of PAH development will rely on the use of many models, we argue that the severe PAH model of Cav1-KO + hypoxia deserves further consideration.

One limitation of this study is that the global Cav1-KO limits our ability to investigate cell-specific transcriptomic changes. Our data reveals how endothelial Cav1 modulation can influence the expression of non-endothelial genes, such as muscle or cardiac differentiation, hinting at Cav1-modulated paracrine signaling in maintaining cell identity. This notion can be further explored using single-cell RNA-sequencing and EC-specific Cav1 knockout models, which our group is currently working towards.

Another limitation is elucidating a mechanism for the substantial sex differences seen in our data and in human patients. The most obvious mechanisms to investigate would either be gene expression from sex chromosomes or hormone-induced transcriptomic changes. While the field has thoroughly documented the sex differences in disease risk and outcome, the underlying mechanism for this difference is another PAH mystery. Combining our Cav1-KO + hypoxia model with other models for investigating sex differences, such as the Four Core genotype (De Vries et al., 2002; Arnold and Chen, 2009) or gonadectomies, may be useful in elucidating this question and will be incorporated into future investigations.

Based on our observations, the strongest candidates for transcriptomic pathways with unique sex dynamics in PAH development would be angiogenesis and muscle cell development. In our experiments, female mice demonstrated more significant changes to angiogenic signaling than males, particularly the downregulation seen in the female Cav1-KO hypoxia group. Dysregulated angiogenic signaling and aberrant angiogenesis are key hallmarks of PAH (Pathology and pathobiology of pulmonary hypertension: state of the art and research perspectives, 2019; Southgate et al., 2020), and our data suggests more substantial transcriptomic changes to angiogenic signaling in experimental females than in males. Key genes uniquely downregulated in female Cav1-KO hypoxia are *cdh5, vegfd*, and *mmp2.* This sex-specific dysregulation of angiogenic signaling may contribute to aberrant angiogenesis and may explain increased female susceptibility to PAH in human patients, which deserves extra investigation. Changes to BMP signaling may also underly the changes in angiogenic signaling, though in that case there was also male-specific upregulation in Cav1-RC.

Similarly, muscle cell development showed more substantial transcriptomic changes in the male experimental mice compared to the female groups, and smooth muscle hypertrophy has been identified as a physiological hallmark of PAH development (Pathology and pathobiology of pulmonary hypertension: state of the art and research perspectives, 2019; Southgate et al., 2020; Ruopp and Cockrill, 2022). In our data, the genes uniquely upregulated in males were *myom2, myoz2*, and *csrp3.* While there are also changes to muscle cell development seen in female RC mice, they are more significantly altered in male mice, particularly in Cav1-KO. In combination with data showing increased mortality in chronic hypoxia, this dysregulation of muscle cell development may lead to the muscle cell hypertrophy seen in PAH patients and the increased mortality rate of male PAH patients, which deserves further investigation. *Myom2* is found upregulated in both male Cav1-KO and in both sexes of Cav1-RC, whereas *myoz2* and *csrp3* were not upregulated in both sexes of Cav1-RC like they were in Cav1-KO males. Thus, investigating these genes and pathways and their sex dynamic during hypoxia in caveolin depleted conditions may illuminate how these subtle differences lead to substantial differences in PAH outcome.

Ultimately, this study aimed to uncover sex-specific physiological and transcriptomic changes that underly the development of PAH induced by hypoxia and Cav-1 depletion. The reduction of hypoxia-induced mortality in Cav1-RC mice clearly demonstrated the vital role that endothelial Cav1 expression plays in preventing PAH development, and thus we investigated the underlying transcriptomics. While many of the pathways known to contribute to PAH development were found in both males and females (BMP signaling, cilia organization, muscle cell development, etc.), the degree of transcriptional change, specific genes within pathways, and the exact conditions that induced the change were not identical. Clearly PAH and its unique sex dynamic do not come from any single factor, and thus fully investigating it will require a fully nuanced understanding of these pathways and how they are regulated.

## Methods

### Mouse experiments

Cav1^-/-^ (Cav1^tm1Mls^/J) and B6/129SJ2 wild-type control mice were originally purchased from Jackson Labs. Cav1-RC mice (Murata et al., 2007) were obtained from William Sessa, Yale University. All animal experiments were approved by the Institutional Animal Care and Use Committee of the University of Illinois Chicago.

*Chronic Hypoxia Exposure.* Mice were exposed to hypoxic conditions via a hypoxia chamber that utilizes nitrogen gas to displace oxygen to a final concentration of 10%. This level is maintained by an automatic electronic oxygen meter, keeping the approximate oxygen gas content at 10%. This was maintained for 8 weeks in the case of survival studies, or 6 weeks for tissue isolation and echocardiography. The mice were observed daily for symptoms such as pain, distress, lethargy, or other signs of ill health, at which point they were humanely euthanized.

### Animal models of pulmonary arterial hypertension and hemodynamic measurements

In the mouse model of hypoxia-mediated pulmonary arterial hypertension, the mice mentioned above were exposed to hypoxia (10% O_2_) in a ventilated chamber (Biospherix, New York) for up to 6 weeks. Following exposure, right ventricular systolic pressure (RVSP) was determined by right heart catheterization using a Millar pressure transducer catheter. Fulton index, a weight ratio of the right ventricle divided by the sum of left ventricle and septum (RV/(LV+S)), was measured to determine the extent of right ventricular hypertrophy (RVH). 

*Rodent echocardiography.* Mouse heart function was assessed using functional rodent echocardiography, following previously described procedures. Briefly, the animals were anesthetized with inhaled isoflurane administered through a nose cone. Transthoracic echo was performed using the VisualSonics Vevo 2100 (VisualSonics Inc., Toronto, ON, Canada) and MS-550D transducer. RVFWT was measured during end-diastole at the parasternal short-axis mitral valve level using two-dimensional (2D) imaging or at the parasternal long-axis RV outflow tract level using M-mode imaging. Pulse-wave Doppler echo was utilized to record pulmonary blood outflow at the level of the aortic valve in the long-axis view, enabling measurement of PAT and PET. TAPSE was measured in 2D M-mode echocardiograms obtained from the apical four-chamber view. The cursor was positioned on the lateral tricuspid annulus near the free RV wall, aligning it as closely as possible to the apex of the heart.

### Left ventricular measurements

LV systolic functions were assessed using M-mode echocardiograms obtained from the LV parasternal short-axis view, recorded at the level of the two papillary muscles. Calculation of LV ejection fraction (EF), cardiac output (CO), stroke volume (SV), and fractional shortening (FS) normalized by body weight was performed. LV diastolic functions were evaluated from apical 4-chamber views. Doppler-mode echocardiograms were used to acquire mitral flow velocity, including peak early (E) and atrial (A) velocities, as well as isovolumic relaxation time (IVRT). Additionally, left ventricular global longitudinal strain (LV GLS), an important clinical parameter indicating heart recovery from heart failure, was measured using strain imaging analysis of LV B-mode videos. All the aforementioned parameters were averaged over three cardiac cycles.

### Blood collection for gas measurement

For each mouse, approximately 100 μL of blood was extracted from the anterior vena cava using a sterile syringe without the use of anti-coagulant. The samples were then injected into individual i-STAT CG4+ cartridges (Abbott, #03P85-51) to approximately the recommended 95 μL volume. Cartridges were placed into an i-STAT 1 machine (Abbott, #04P7501) for automated analysis of blood gases.

*Western blot.* Lung tissue was homogenized in lysis buffer [(Tris buffer pH 7.5, 150 nM NaCI, 1 mM NaF, 1mM EDTA, 1 mM Na3VO4, 44 µg/ml phenylmethylsulfonyl fluoride [PMSF], 1% protease inhibitor cocktail and 2% ODG)] using a tissue homogenizer. Samples were then centrifuged for 20 minutes at 16, 000 x g at 4 °C followed by protein quantification using a Pierce BCA protein assay kit (Thermoscientific #23225). Samples were prepared in 4 x sample buffer (Biorad, #1610747) at a concentration of 50 ug per well with heat denaturation. Samples were then loaded onto midi size TGX stain-free gels (4-15%) followed by transfer onto a low fluorescence PVDF membranes using a turbo-transfer system. After 1 hr block with skim milk at room temperature (RT), samples were incubated with mouse Caveolin-1 (BD Biosciences, #610407) overnight at 4 °C. After washing with tris-based saline with 0.05% Tween, membranes were incubated with anti-mouse-horse radish peroxidase (HRP) for 1 hr at RT. A chemi-doc Biorad imager in conjunction with ECL-max was used to develop the membranes.

### RNA sequencing

Whole lungs were collected from experimental mice and whole RNA was extracted using TRIzol reagent (Invitrogen, #15596026) with chloroform extraction. RNA was shipped to Novagene (Sacramento, CA) who processed the samples for sequencing. Samples underwent quality control and RNA integrity number (RIN) led to one sample being excluded from sequencing and analysis. For library construction, messenger RNA was purified from total RNA by poly-A enrichment and reverse transcribed to construct directional libraries. NovaSeq 6000 PE150 was used for paired-end sequencing of libraries. Sequences were mapped to mus musculus reference genome mm10 using hisat2 2.0.5 with default parameters. Read numbers and fragments per kilobase of transcript per million mapped reads (FPKM) were calculated using featureCounts 1.5.0-p3 with default parameters. Each group contains n=3 mice except female Cav1-KO normoxia and female Cav1-RC normoxia which contain n=2.

Quantitative PCR. Lung RNA was isolated using standard TRIzol reagent (Invitrogen, #15596026) with chloroform extraction. First, lung tissue was preserved in RNAlater (Invitrogen, #AM7020) during tissue collection and processed with TRIzol using a tissue homogenizer. RNA was eluted using RNAse free water and quantified RNA was reverse-transcribed into cDNA with High-Capacity cDNA Reverse Transcription Kit (ThermoFisher Scientific, #4368814). RT-qPCR was performed using FastStart Universal SYBR Green Master mix (Sigma Aldrich, #4913914001) with the following primers:

*BMPR2* Forward: 5’ CTGGCCATGATGAAGGGGTT 3’.
*BMPR2* Reverse: 5’ AGAATTGGGCCTCTGTGCTC 3’.
*DKK2* Forward: 5’ GACAACTCCTAACCGCACCA 3’.
*DKK2* Reverse: 5’ CTCCTCTGCTCCTCTGTCCT 3’.
*eNOS* Forward: 5’ ATGTGCTGCCCCTGTTACTC 3’.
*eNOS* Reverse: 5’ CACAGGTCCCTCATGCCAAT 3’.
*RBPJ* Forward: 5’ TTCAGTTGTGGTGTGTGGCT 3’.
*RBPJ* Reverse: 5’ GGCCTGCCTTCCAATCTCTT 3’.
*Actin Beta* Forward: 5’ GGCTGTATTCCCCTCCATCG 3’.
*Actin Beta* Reverse: 5’ CCAGTTGGTAACAATGCCATGT 3’.

### Differential gene expression and gene ontology analysis

DEG and GO analysis was performed using the Novomagic platform from Novogene. Pairwise comparison of treatment/genotype groups were performed with Deseq2 1.20.0. Genes were considered differentially expressed when |log_2_fold change > 1| and the adjusted p-value (or P_adj_) < 0.05. Heatmaps, bar graphs, and PCA chart were generated using previously developed R packages (Love et al., 2014; Wickham, 2016; Sievert, 2020; Neuwirth, 2022; Ali, 2024; Chang, 2025; Kolde, 2025; Vaidyanathan et al., 2025; Wickham and Bryan, 2025; Wickham et al., 2025). Heatmaps of DEGs shown were generated using normalized FPKM expression values. These DEGs were input to clusterProfiler 3.8.1 for GO analysis and threshold was set to P_adj_ < 0.05.

### Statistical analysis

Experimental data is shown here as the mean value and +/- standard error mean (SEM) for experimentally independent replicates. Three-way ANOVA was performed with sex, hypoxia status, and genotype as the three factors. Multiple comparisons analysis was performed between every group using Bonferonni’s multiple comparisons test. Significant comparisons between groups that differ by a single factor are shown in each graph analyzed by three-way ANOVA. Data was analyzed using Graphpad Prism 10. Significance of comparisons are shown using standard demarcation: *p < 0.05, **p < 0.01, ***p < 0.001, ****p < 0.0001, and (not significant) *ns p* > 0.05.

## Data Availability

The datasets presented in this study can be found in online repositories. The names of the repository/repositories and accession number(s) can be found below: https://www.ncbi.nlm.nih.gov/geo/, GSE316502.
