# Supplementary material for: Transcriptomic profiling of the sex-linked biological pathways of severe pulmonary arterial hypertension associated with endothelial cell caveolin-1 depletion and chronic hypoxia
Source: Front Physiol. 2026 May 8;17:1794886. doi: 10.3389/fphys.2026.1794886 (PMC13195401; doi:10.3389/fphys.2026.1794886)
Supplement: Supplementary file 1 [file Presentation1.pptx]

## Slide 1
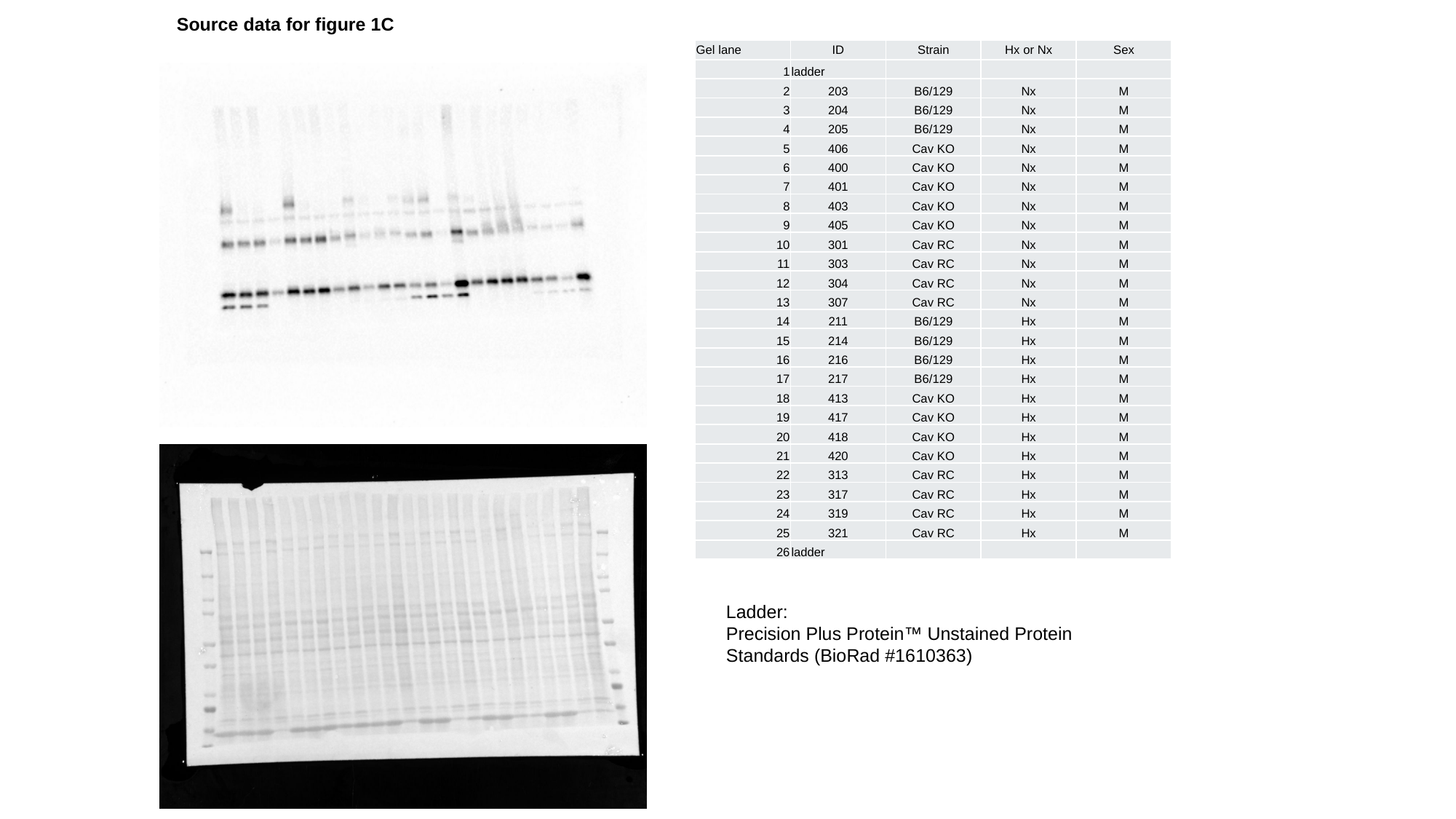

Source data for figure 1C
| Gel lane | ID | Strain | Hx or Nx | Sex |
| --- | --- | --- | --- | --- |
| 1 | ladder | | | |
| 2 | 203 | B6/129 | Nx | M |
| 3 | 204 | B6/129 | Nx | M |
| 4 | 205 | B6/129 | Nx | M |
| 5 | 406 | Cav KO | Nx | M |
| 6 | 400 | Cav KO | Nx | M |
| 7 | 401 | Cav KO | Nx | M |
| 8 | 403 | Cav KO | Nx | M |
| 9 | 405 | Cav KO | Nx | M |
| 10 | 301 | Cav RC | Nx | M |
| 11 | 303 | Cav RC | Nx | M |
| 12 | 304 | Cav RC | Nx | M |
| 13 | 307 | Cav RC | Nx | M |
| 14 | 211 | B6/129 | Hx | M |
| 15 | 214 | B6/129 | Hx | M |
| 16 | 216 | B6/129 | Hx | M |
| 17 | 217 | B6/129 | Hx | M |
| 18 | 413 | Cav KO | Hx | M |
| 19 | 417 | Cav KO | Hx | M |
| 20 | 418 | Cav KO | Hx | M |
| 21 | 420 | Cav KO | Hx | M |
| 22 | 313 | Cav RC | Hx | M |
| 23 | 317 | Cav RC | Hx | M |
| 24 | 319 | Cav RC | Hx | M |
| 25 | 321 | Cav RC | Hx | M |
| 26 | ladder | | | |
Ladder:
Precision Plus Protein™ Unstained Protein Standards (BioRad #1610363)

## Slide 2
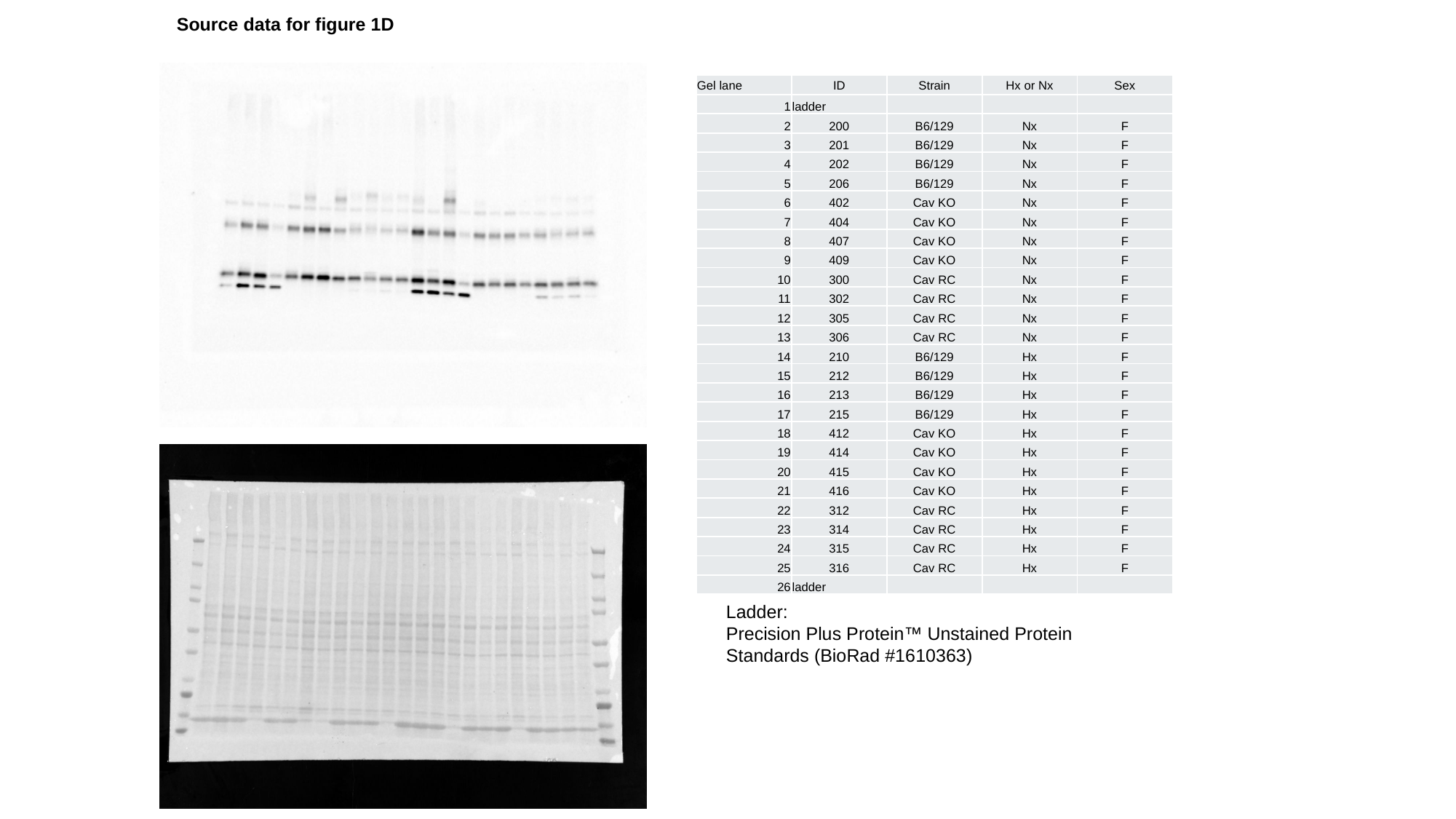

Source data for figure 1D
| Gel lane | ID | Strain | Hx or Nx | Sex |
| --- | --- | --- | --- | --- |
| 1 | ladder | | | |
| 2 | 200 | B6/129 | Nx | F |
| 3 | 201 | B6/129 | Nx | F |
| 4 | 202 | B6/129 | Nx | F |
| 5 | 206 | B6/129 | Nx | F |
| 6 | 402 | Cav KO | Nx | F |
| 7 | 404 | Cav KO | Nx | F |
| 8 | 407 | Cav KO | Nx | F |
| 9 | 409 | Cav KO | Nx | F |
| 10 | 300 | Cav RC | Nx | F |
| 11 | 302 | Cav RC | Nx | F |
| 12 | 305 | Cav RC | Nx | F |
| 13 | 306 | Cav RC | Nx | F |
| 14 | 210 | B6/129 | Hx | F |
| 15 | 212 | B6/129 | Hx | F |
| 16 | 213 | B6/129 | Hx | F |
| 17 | 215 | B6/129 | Hx | F |
| 18 | 412 | Cav KO | Hx | F |
| 19 | 414 | Cav KO | Hx | F |
| 20 | 415 | Cav KO | Hx | F |
| 21 | 416 | Cav KO | Hx | F |
| 22 | 312 | Cav RC | Hx | F |
| 23 | 314 | Cav RC | Hx | F |
| 24 | 315 | Cav RC | Hx | F |
| 25 | 316 | Cav RC | Hx | F |
| 26 | ladder | | | |
Ladder:
Precision Plus Protein™ Unstained Protein Standards (BioRad #1610363)

## Slide 3
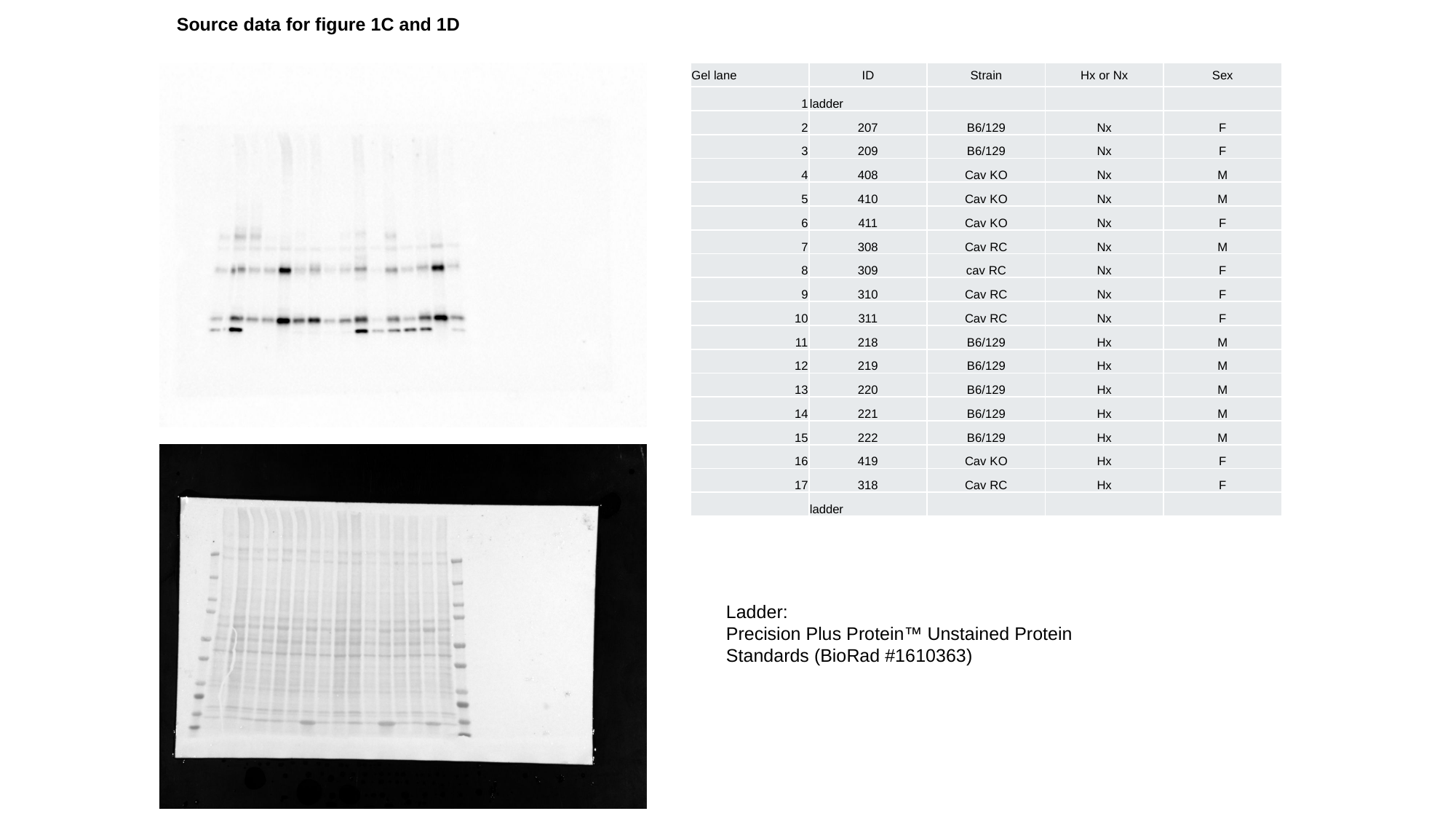

Source data for figure 1C and 1D
| Gel lane | ID | Strain | Hx or Nx | Sex |
| --- | --- | --- | --- | --- |
| 1 | ladder | | | |
| 2 | 207 | B6/129 | Nx | F |
| 3 | 209 | B6/129 | Nx | F |
| 4 | 408 | Cav KO | Nx | M |
| 5 | 410 | Cav KO | Nx | M |
| 6 | 411 | Cav KO | Nx | F |
| 7 | 308 | Cav RC | Nx | M |
| 8 | 309 | cav RC | Nx | F |
| 9 | 310 | Cav RC | Nx | F |
| 10 | 311 | Cav RC | Nx | F |
| 11 | 218 | B6/129 | Hx | M |
| 12 | 219 | B6/129 | Hx | M |
| 13 | 220 | B6/129 | Hx | M |
| 14 | 221 | B6/129 | Hx | M |
| 15 | 222 | B6/129 | Hx | M |
| 16 | 419 | Cav KO | Hx | F |
| 17 | 318 | Cav RC | Hx | F |
| | ladder | | | |
Ladder:
Precision Plus Protein™ Unstained Protein Standards (BioRad #1610363)
