# Supplementary material for: Transcriptomic profiling of the sex-linked biological pathways of severe pulmonary arterial hypertension associated with endothelial cell caveolin-1 depletion and chronic hypoxia
Source: Front Physiol. 2026 May 8;17:1794886. doi: 10.3389/fphys.2026.1794886 (PMC13195401; doi:10.3389/fphys.2026.1794886)
Supplement: Supplementary file 2 [file Supplementaryfile2.pptx]

## Slide 1
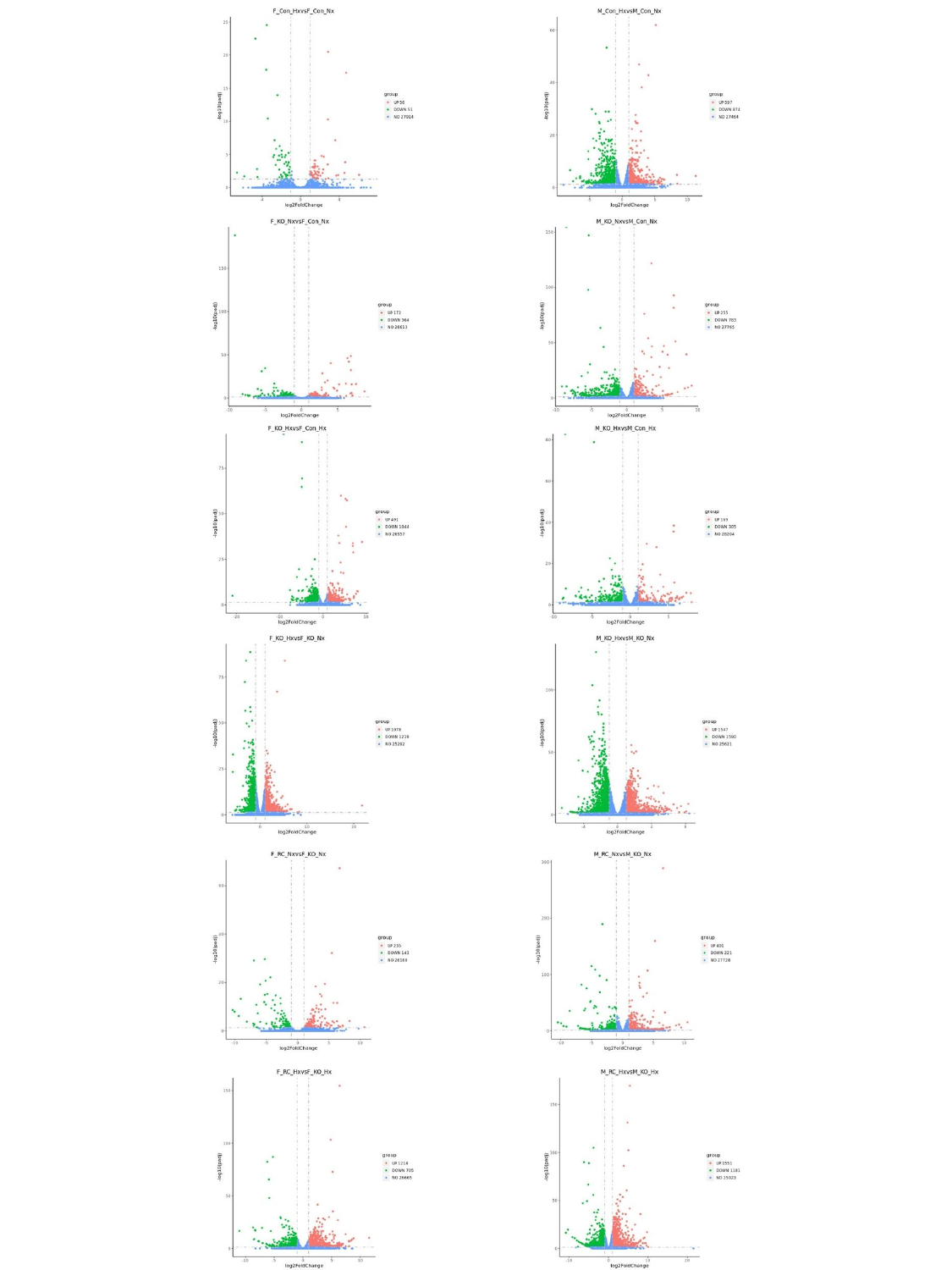

## Slide 2
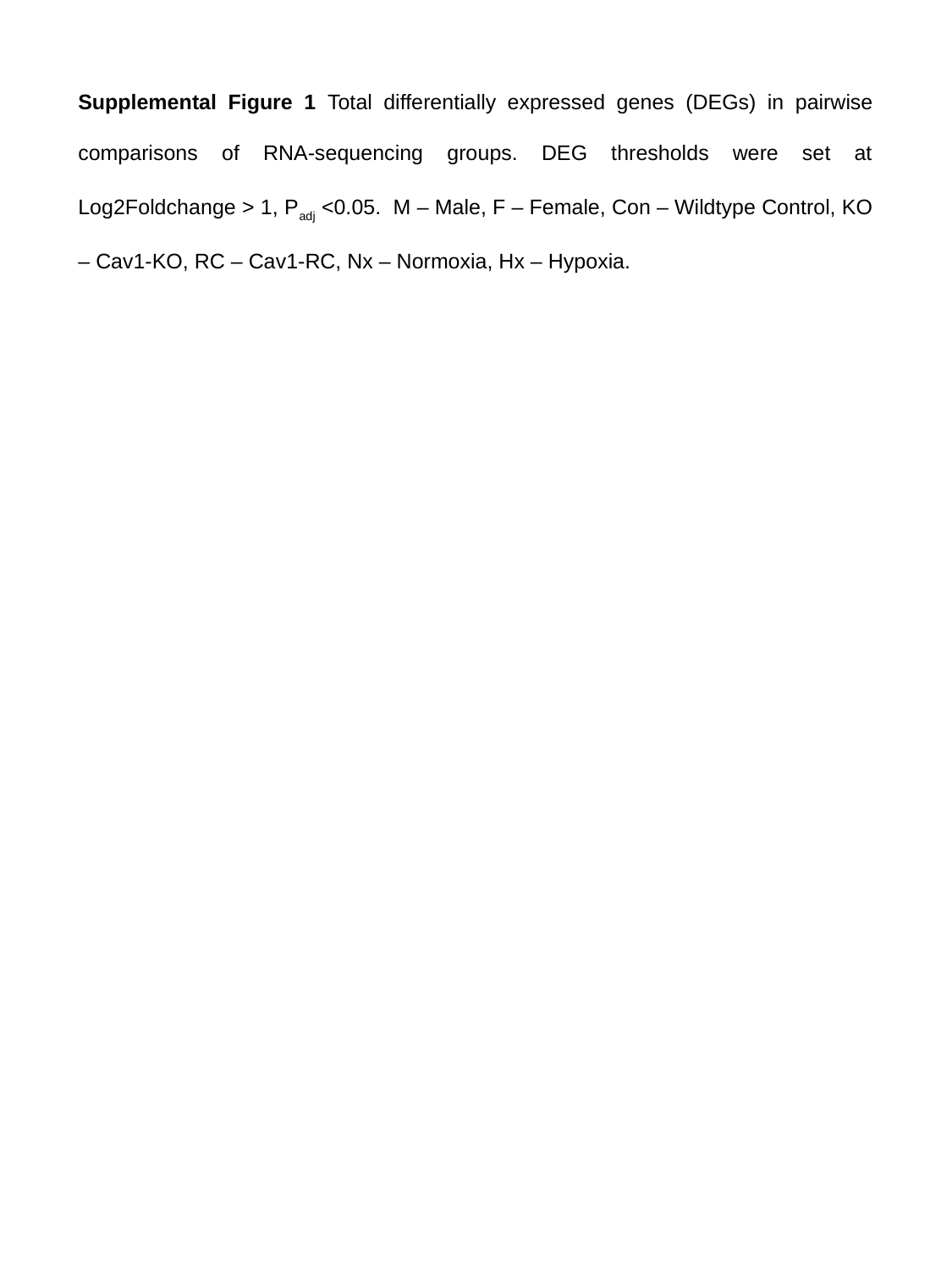

Supplemental Figure 1 Total differentially expressed genes (DEGs) in pairwise comparisons of RNA-sequencing groups. DEG thresholds were set at Log2Foldchange > 1, Padj <0.05. M – Male, F – Female, Con – Wildtype Control, KO – Cav1-KO, RC – Cav1-RC, Nx – Normoxia, Hx – Hypoxia.

## Slide 3
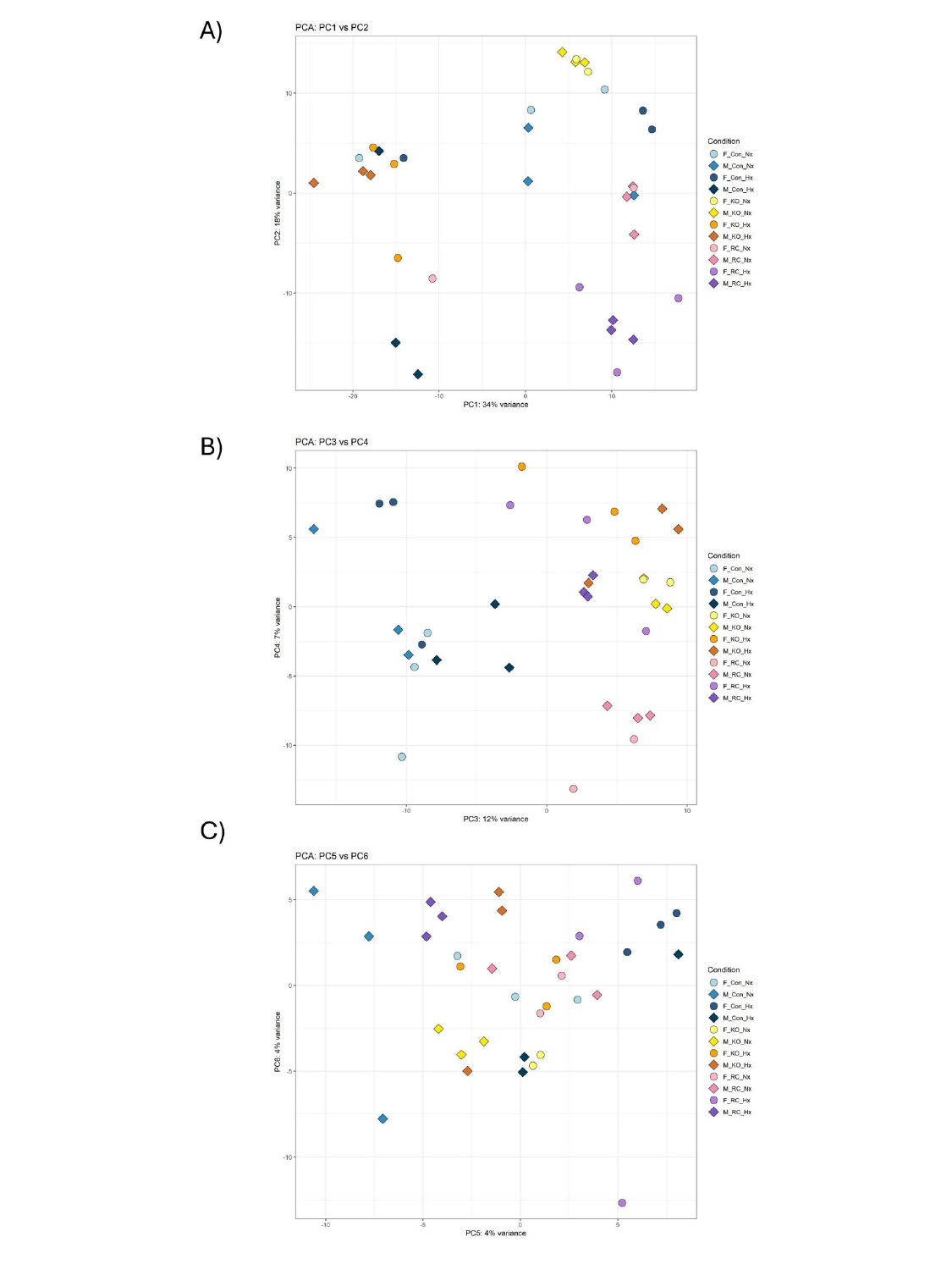

## Slide 4
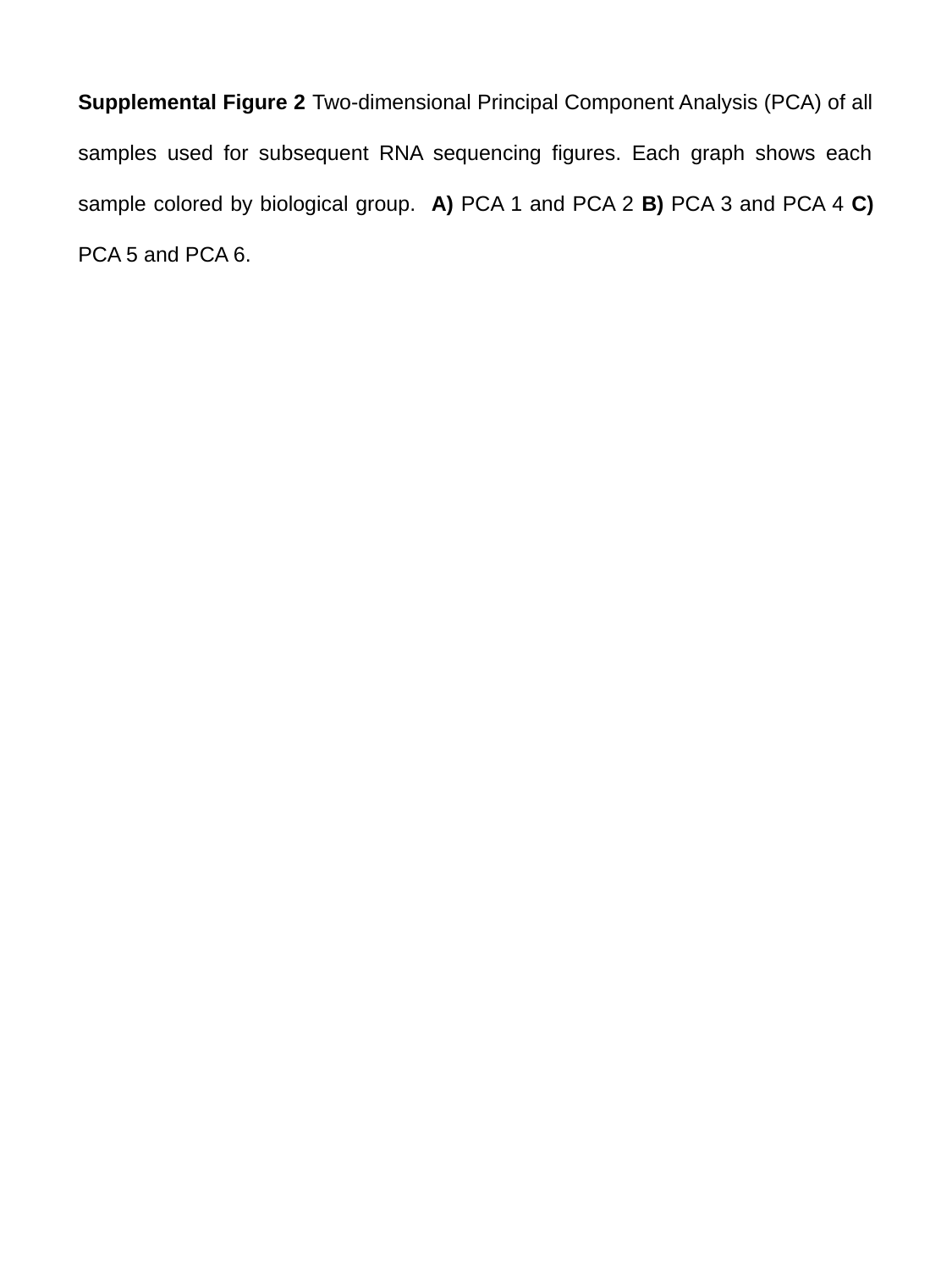

Supplemental Figure 2 Two-dimensional Principal Component Analysis (PCA) of all samples used for subsequent RNA sequencing figures. Each graph shows each sample colored by biological group. A) PCA 1 and PCA 2 B) PCA 3 and PCA 4 C) PCA 5 and PCA 6.

## Slide 5
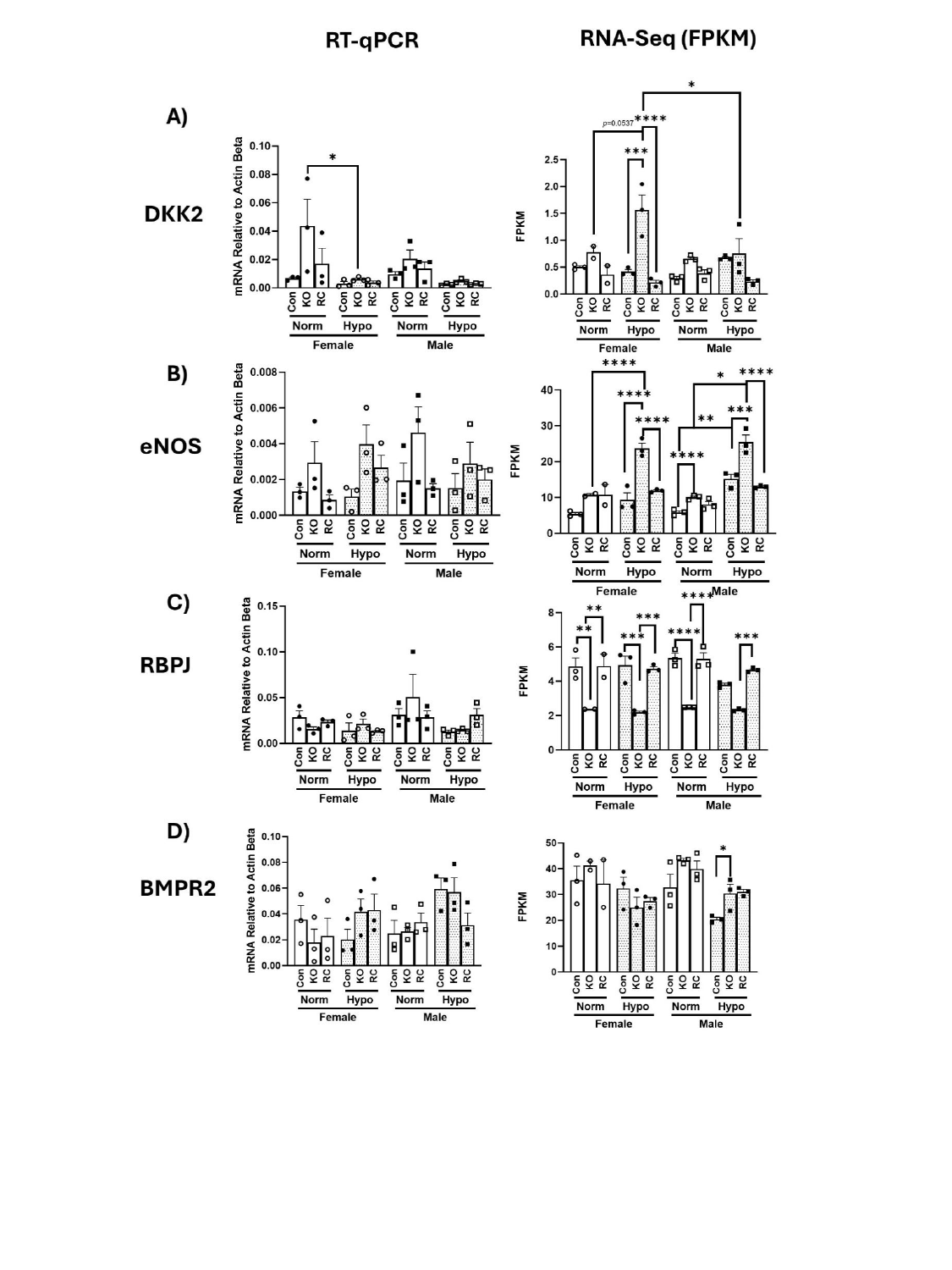

## Slide 6
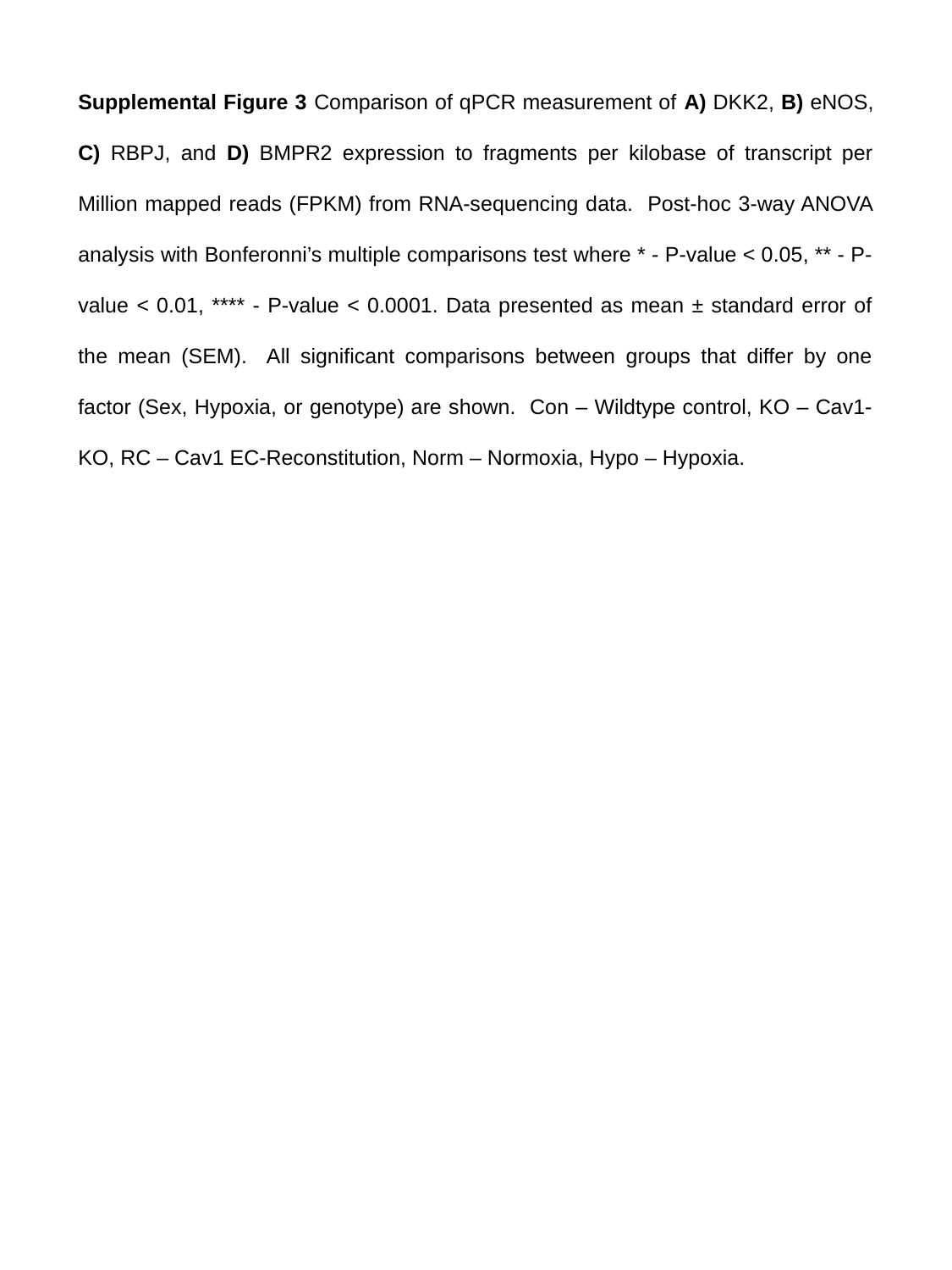

Supplemental Figure 3 Comparison of qPCR measurement of A) DKK2, B) eNOS, C) RBPJ, and D) BMPR2 expression to fragments per kilobase of transcript per Million mapped reads (FPKM) from RNA-sequencing data. Post-hoc 3-way ANOVA analysis with Bonferonni’s multiple comparisons test where * - P-value < 0.05, ** - P-value < 0.01, **** - P-value < 0.0001. Data presented as mean ± standard error of the mean (SEM). All significant comparisons between groups that differ by one factor (Sex, Hypoxia, or genotype) are shown. Con – Wildtype control, KO – Cav1-KO, RC – Cav1 EC-Reconstitution, Norm – Normoxia, Hypo – Hypoxia.

## Slide 7
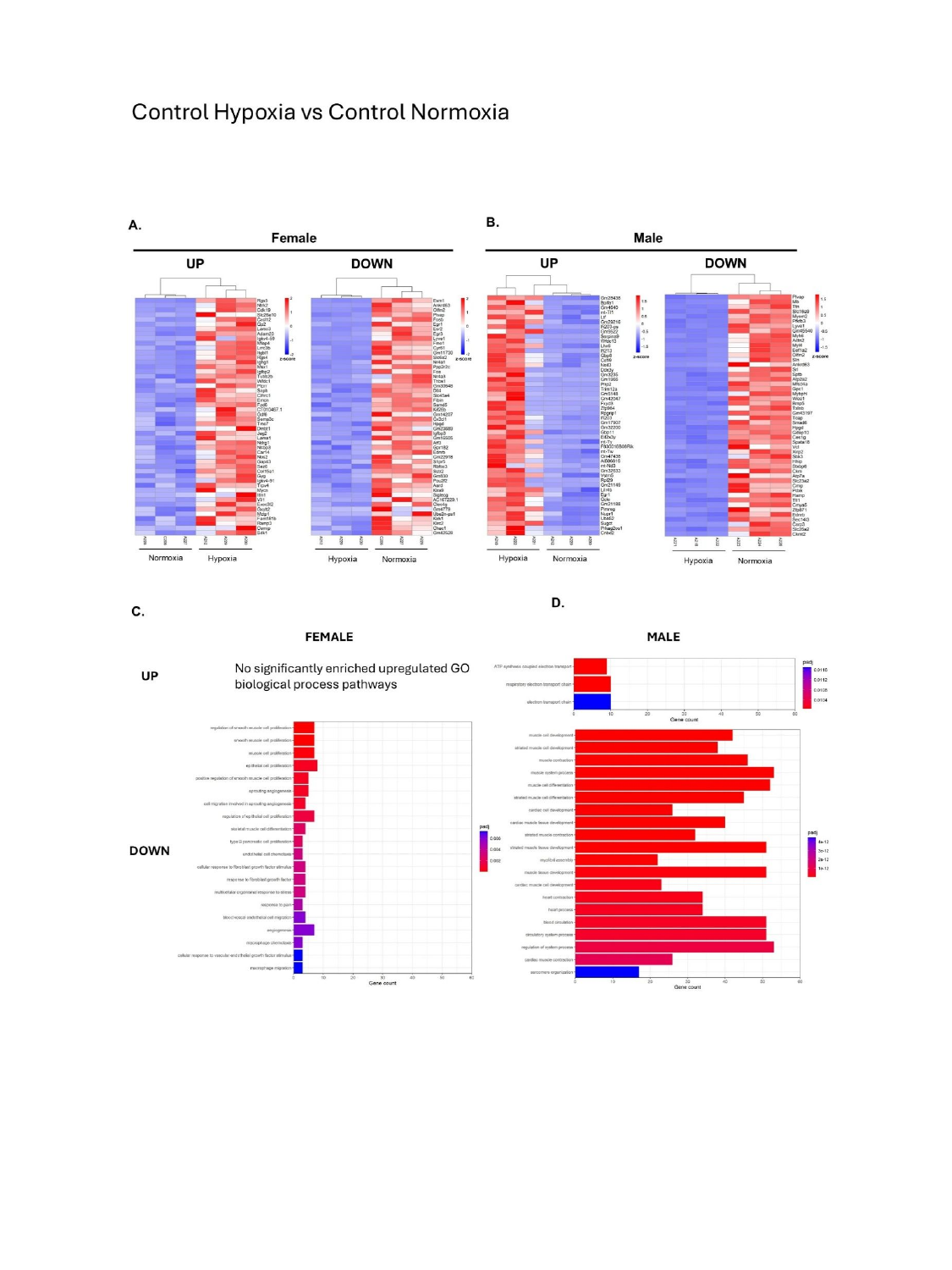

## Slide 8
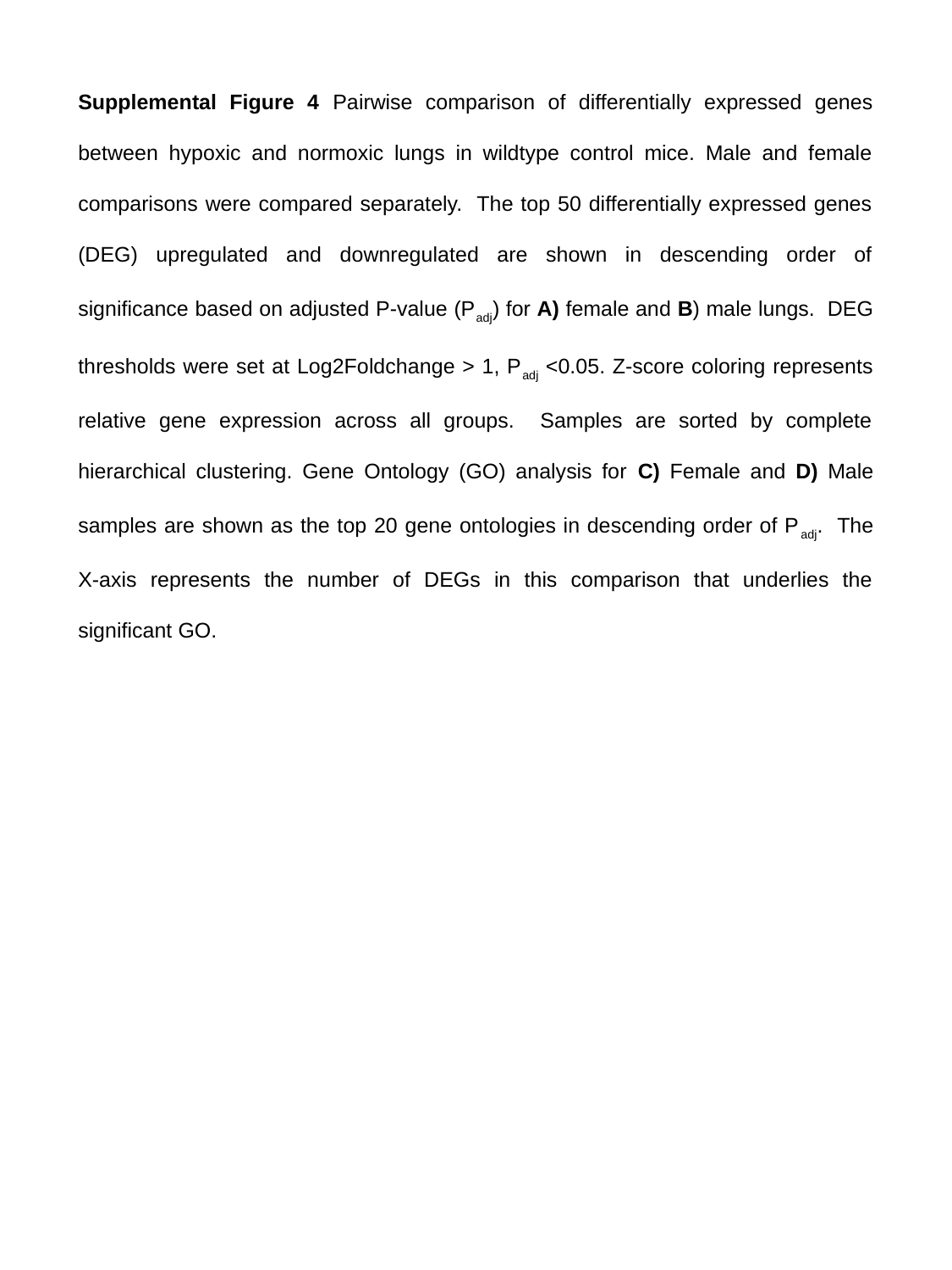

Supplemental Figure 4 Pairwise comparison of differentially expressed genes between hypoxic and normoxic lungs in wildtype control mice. Male and female comparisons were compared separately. The top 50 differentially expressed genes (DEG) upregulated and downregulated are shown in descending order of significance based on adjusted P-value (Padj) for A) female and B) male lungs. DEG thresholds were set at Log2Foldchange > 1, Padj <0.05. Z-score coloring represents relative gene expression across all groups. Samples are sorted by complete hierarchical clustering. Gene Ontology (GO) analysis for C) Female and D) Male samples are shown as the top 20 gene ontologies in descending order of Padj. The X-axis represents the number of DEGs in this comparison that underlies the significant GO.

## Slide 9
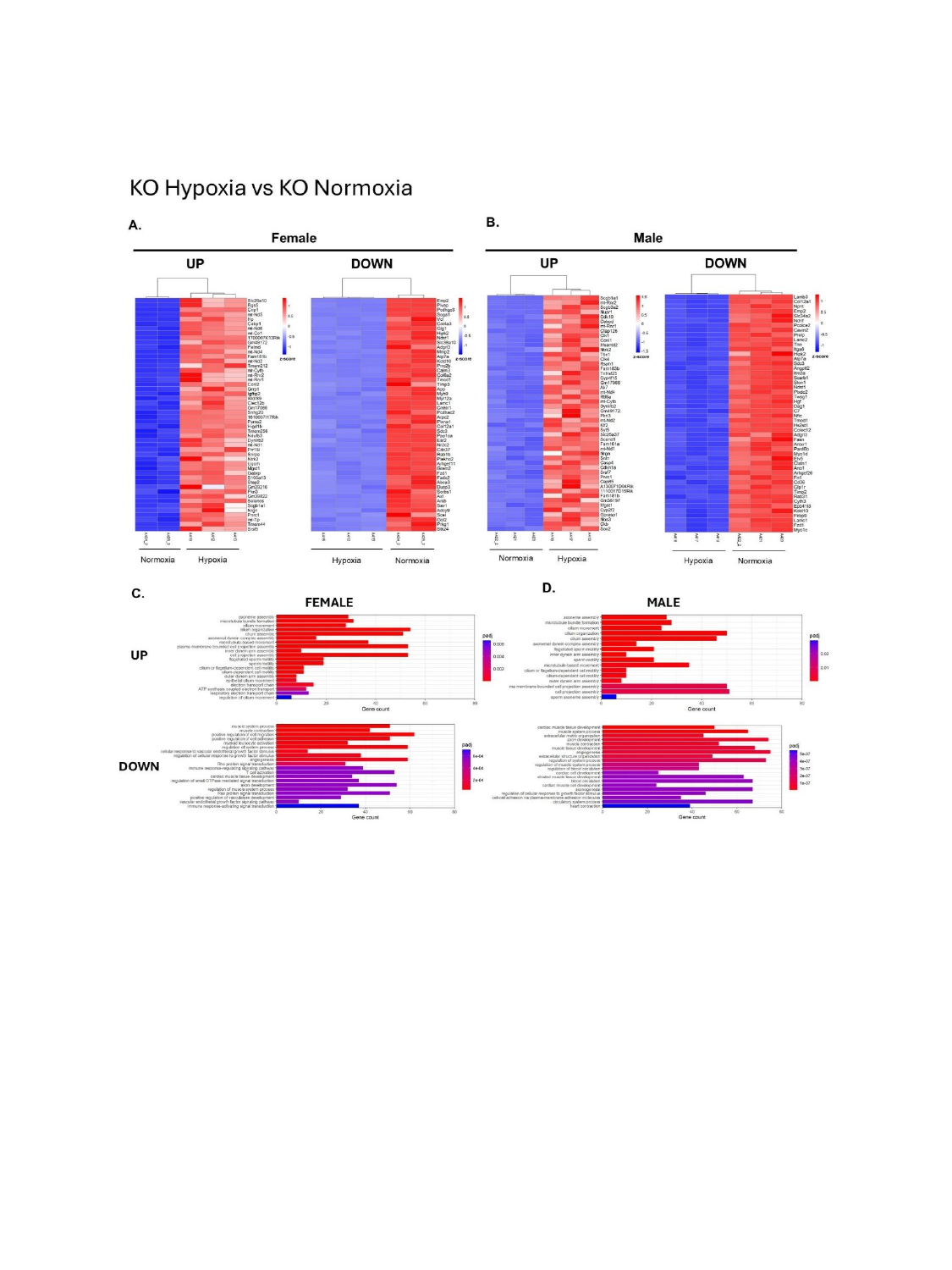

## Slide 10
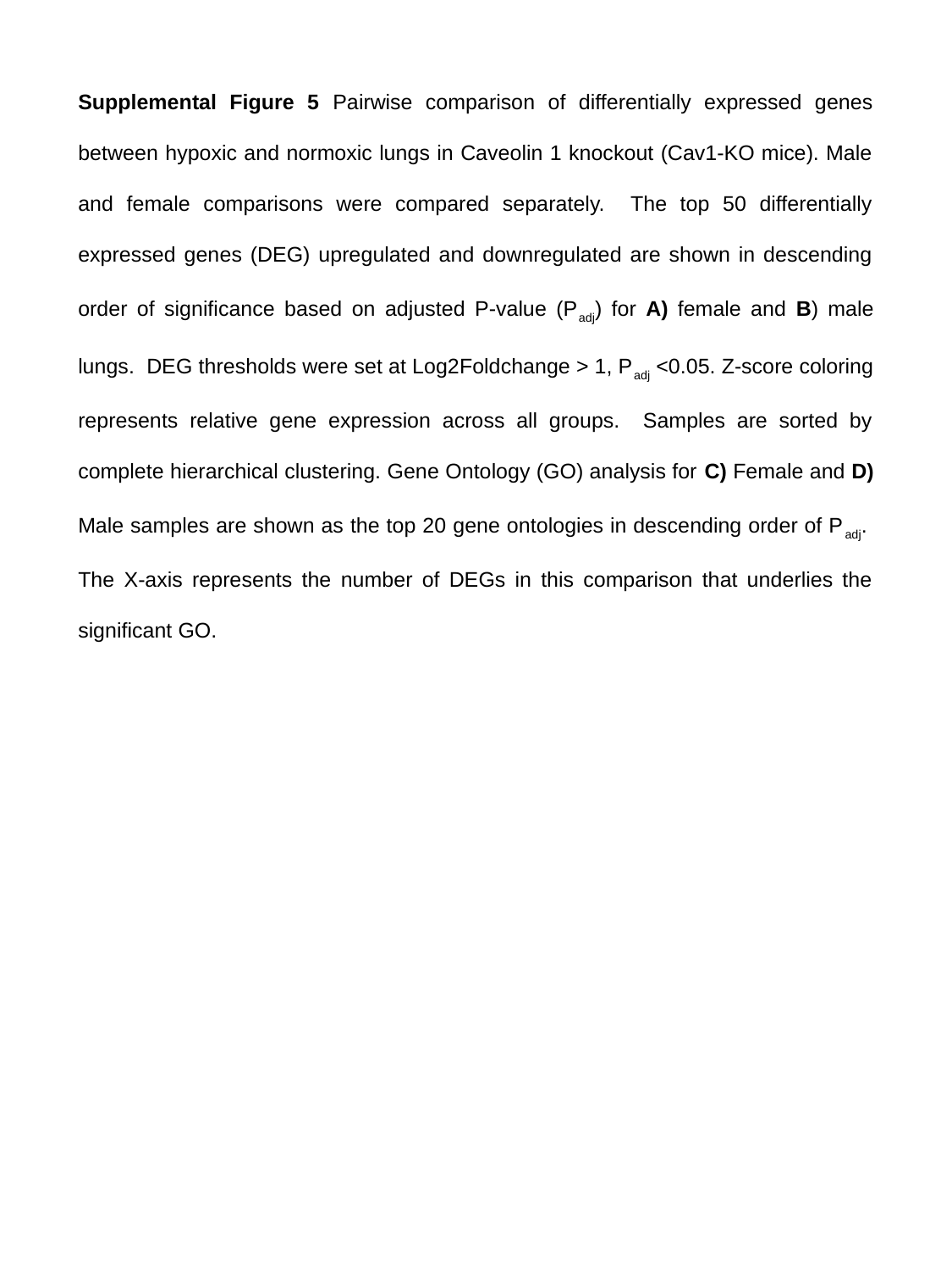

Supplemental Figure 5 Pairwise comparison of differentially expressed genes between hypoxic and normoxic lungs in Caveolin 1 knockout (Cav1-KO mice). Male and female comparisons were compared separately. The top 50 differentially expressed genes (DEG) upregulated and downregulated are shown in descending order of significance based on adjusted P-value (Padj) for A) female and B) male lungs. DEG thresholds were set at Log2Foldchange > 1, Padj <0.05. Z-score coloring represents relative gene expression across all groups. Samples are sorted by complete hierarchical clustering. Gene Ontology (GO) analysis for C) Female and D) Male samples are shown as the top 20 gene ontologies in descending order of Padj. The X-axis represents the number of DEGs in this comparison that underlies the significant GO.
